# Modelling and Control of a 2-DOF Robot Arm with Elastic Joints for Safe Human-Robot Interaction

**DOI:** 10.3389/frobt.2021.679304

**Published:** 2021-08-18

**Authors:** Hua Minh Tuan, Filippo Sanfilippo, Nguyen Vinh Hao

**Affiliations:** ^1^ Department of Control Engineering and Automation, Faculty of Electrical and Electronic Engineering, Ho Chi Minh City University of Technology (HCMUT), Ho Chi Minh City, Vietnam; ^2^ Vietnam National University Ho Chi Minh City, Ho Chi Minh City, Vietnam; ^3^ Department of Engineering Sciences, University of Agder (UiA), Grimstad, Norway

**Keywords:** collaborative robot, robot arm, series elastic actuator, human-robot interaction, robotics

## Abstract

Collaborative robots (or cobots) are robots that can safely work together or interact with humans in a common space. They gradually become noticeable nowadays. Compliant actuators are very relevant for the design of cobots. This type of actuation scheme mitigates the damage caused by unexpected collision. Therefore, elastic joints are considered to outperform rigid joints when operating in a dynamic environment. However, most of the available elastic robots are relatively costly or difficult to construct. To give researchers a solution that is inexpensive, easily customisable, and fast to fabricate, a newly-designed low-cost, and open-source design of an elastic joint is presented in this work. Based on the newly design elastic joint, a highly-compliant multi-purpose 2-DOF robot arm for safe human-robot interaction is also introduced. The mechanical design of the robot and a position control algorithm are presented. The mechanical prototype is 3D-printed. The control algorithm is a two loops control scheme. In particular, the inner control loop is designed as a model reference adaptive controller (MRAC) to deal with uncertainties in the system parameters, while the outer control loop utilises a fuzzy proportional-integral controller to reduce the effect of external disturbances on the load. The control algorithm is first validated in simulation. Then the effectiveness of the controller is also proven by experiments on the mechanical prototype.

## 1 Introduction

Cobots are robots intended for direct collaborative work with a human operator (Wannasuphoprasit et al., 1997; Peshkin et al., 2001). Cobots appear and support humans in many situations in our daily life, e.g., search and rescue missions (Govindarajan et al., 2016), surveillance and inspection (Donadio et al., 2018; Brito et al., 2020), medical support (Mokaram et al., 2017), etc. One aspect, which makes cobots different from traditional robots, is the capability to mitigate the damage caused by the unpredictable collision in dynamic shared working space. There are various methods to achieve this capability, such as using force/torque sensors (Li et al., 2020), using elastic joints, or using a collision detection algorithm without changing a robot’s physical structure and adding external sensors (Xiao et al., 2018). Using elastic joints is one of the most common methods because it is efficient and low-priced. Conventional actuators are designed by following the traditional principle of “the stiffer the better” (Pratt and Williamson, 1995; Vanderborght et al., 2013). Due to having high force bandwidth, stiff actuators are suitable for position and speed control, and trajectory tracking tasks in isolated environments. However, in unknown environments, these stiff actuators can be damaged by undesired collision. In addition, most low-cost electric motors have to operate at high speed to obtain high torque density. Therefore, to achieve low-speed output for position control tasks, gear reduction is used at the expense of introducing friction, noise, backlash, and torque ripple (Pratt and Williamson, 1995). On the other hand, elastic joints, with compliant motion and shock load absorption characteristics, are developed to tackle these challenges. Shock tolerance of the elastic joints in unstructured environments is enhanced thanks to the elastic components working as a low-pass shock filter. Furthermore, energy storage capability, power output and force sensing are also enhanced (Paine et al., 2015). In spite of numerous advantages, controlling the position and velocity of elastic joints is more challenging than conventional stiff actuators due to the oscillation of elastic components. In addition, disturbance forces can cause the elastic joint to deviate from its original position.

The importance of human-robot interaction cannot be overstated when it comes to cobots, especially when direct physical interaction with humans is considered, i.e., rehabilitation robots. In this perspective, a design of an elastic actuator with springs having different stiffness is introduced in Yu et al. (2013c). The proposed design consists of a servomotor, a ball screw, a torsional spring between the motor and the ball screw, and a set of translational springs between the ball screw nut and the external load. The soft translational springs are used to handle the low force operation and reduce output impedance, stiction, and external shock load. When the translational springs are fully compressed, the torsional spring has a high effective stiffness and enhances the system bandwidth. This design is quite compact. The dynamic modelling and analysis of the proposed actuator is also demonstrated. In (Yu et al. (2013a), this same type of elastic actuator is adopted to design a knee-ankle-foot robot. Successively, a force control approach is presented for the same actuator in Yu et al. (2013b). First, two dynamical actuator methods are introduced based on different force ranges. Second, for the low force range, an optimal control with friction compensation and disturbance rejection, which is augmented by a feedforward control, is presented. The proposed optimal control strategy is further extended to high force ranges. Third, to manage the transition between low and high force control, a switching control technique is provided. The controller is further enhanced in Yu et al. (2015). Human interaction compensation, friction compensation, and a disturbance observer are the primary components of the improved controller. When operating in human-in-charge mode, such a control system allows the robot to achieve low output impedance, while precise force tracking is obtained when operating in force control mode.

**FIGURE 1:**
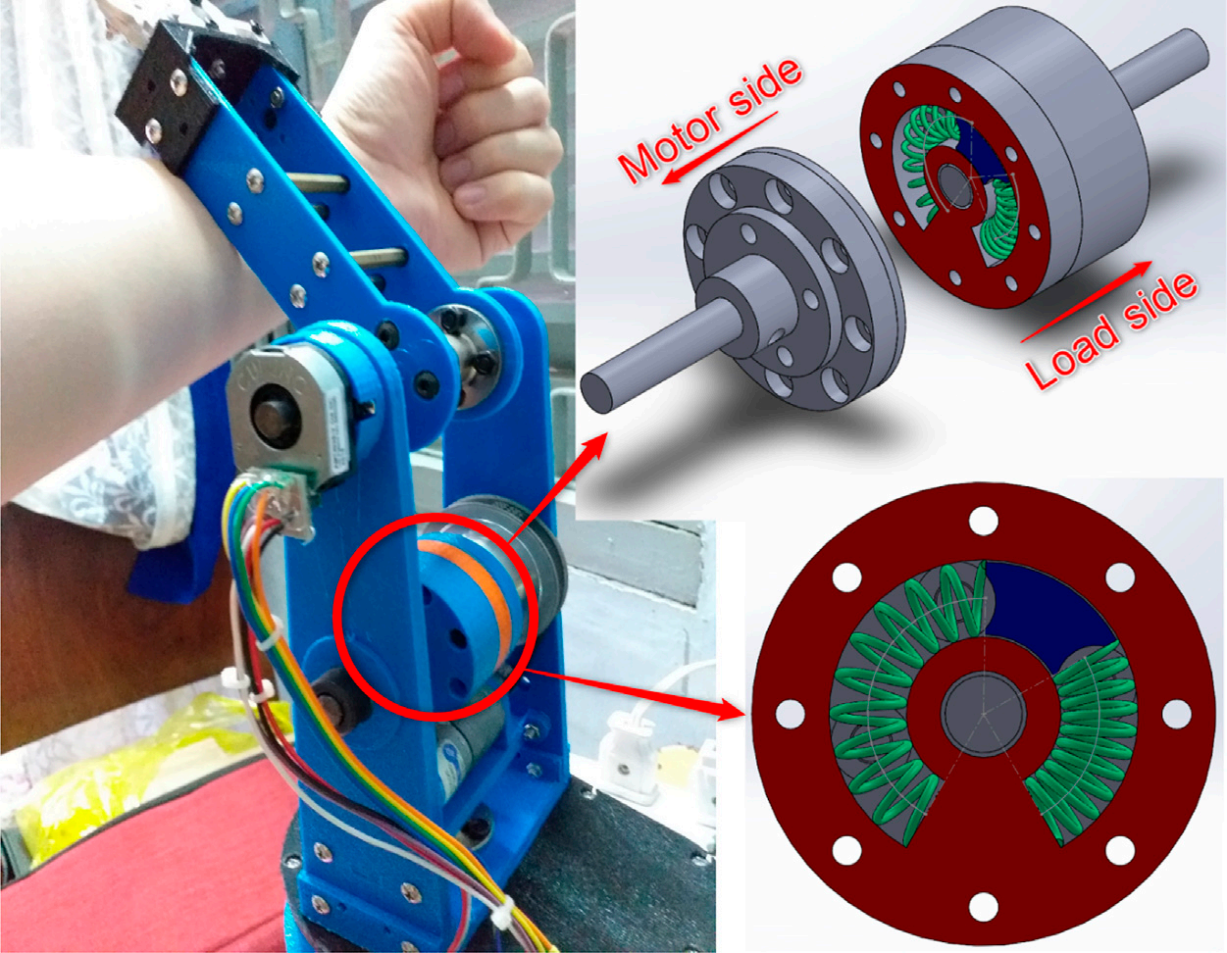
The proposed 2-DOF robot arm with elastic joints for safe human-robot interaction. The real 3D-printed prototype and the 3D model of the joint are shown, respectively.

In line with these research trends, a newly-designed low-cost, open-source design of an elastic joint is proposed in this work to give researchers a solution that is economical, highly adaptable, and quick to manufacture. The design is based on our previously proposed layout for the modules of *Serpens* (Sanfilippo et al., 2019a; Sanfilippo et al., 2019b), a low-cost, open-source and highly compliant multi-purpose modular snake robot. Based on the newly designed elastic joint, the design of a 2-DOF robot arm for safe human-robot interaction is also proposed in this article. The robot components are 3D-printed by using Fused Deposition Modelling (FDM) manufacturing technology, thus making the rapid-prototyping process very economical and quick. The real 3D-printed robot arm is shown in Figure 1, together with the actuator model. Moreover, a decentralised control structure is introduced. Separate controllers are implemented for each joint. The control algorithm is based on a two-feedback loops position control mechanism: an adaptive control is designed in the inner loop to stabilise the system and deal with uncertainties, while a fuzzy controller is considered for the outer loop to eliminate the influence of external force/torque. Then, the controller is validated in simulation with (MATLAB, 2018) and tested in reality with the 3D-printed prototype. The simulation and experiment results show that the decentralised controller could stabilise and precisely control the elastic joint in normal and disturbed conditions.

The paper is organised as follows. A review of the related research work is given in Section 2. In Section 3, we focus on the description of the proposed control algorithm. In Section 4, the physical prototype of the 2-DOF robot arm is presented. In Section 5, related simulation results are outlined. In Section 6, the results of experiments on the physical prototype are shown. Finally, conclusions and future works are discussed in Section 7.

## 2 Related Research Work

### 2.1 Related Control Methods

The advantages of elastic joints are first outlined by Pratt and Williamson’s research (Pratt and Williamson, 1995), where a proportional-integral-derivative (PID) control scheme was proposed. Similarly, proportional-derivative (PD) controllers with on-line gravity compensation are considered in De Luca et al. (2005), Zollo et al. (2007). The global asymptotic stability of these control laws is demonstrated via Lyapunov’s argument and La Salle’s theorem. Another alternative approach is based on robust controllers with a disturbance observer (DOB) (Kim and Chung, 2015). In Talole et al. (2010), a combination of a feedback linearisation-based controller for the trajectory tracking problem and an extended state observer for uncertainty and states estimation is considered. Feedback linearisation and robust integral of sign of error (RISE) methods for controlling position are proposed in Yin et al. (2016). Specifically, the dynamics of the actuator is first feedback linearised, then a RISE method is applied to adapt the system model to uncertainties. A model reference adaptive control approach is implemented in Losey et al. (2016) to adapt to uncertainties in system parameters, while the adoption law is demonstrated using Lyapunov’s theory. In Pérez-Ibarra et al. (2017), a $H_{\infty }$ force controller is used to deliver precisely the force required by a PD position controller on the outer loop. In Kaya and Çetin (2017), an adaptive state feedback regulator and a conventional PI controller is adopted to control a rotary series elastic actuator. The adaptive state feedback regulator define the transient behaviour of the SEA, then, the tracking error is reduced by the PI controller. In Penzlin et al. (2019), a clutched parallel elastic actuator (CPEA) with an iterative learning control loop and a cascade control loop is proposed. The cascade control loop is used to control the position output of a BLDC motor. The iterative learning control loop is used to control the output trajectory of the actuator. To the best of our knowledge, an adaptive control aiming at both stabilising the system as well as dealing with uncertainties to eliminate the influence of external force/torque has not been released yet.

### 2.2 Related Mechanical Design

**FIGURE 2:**
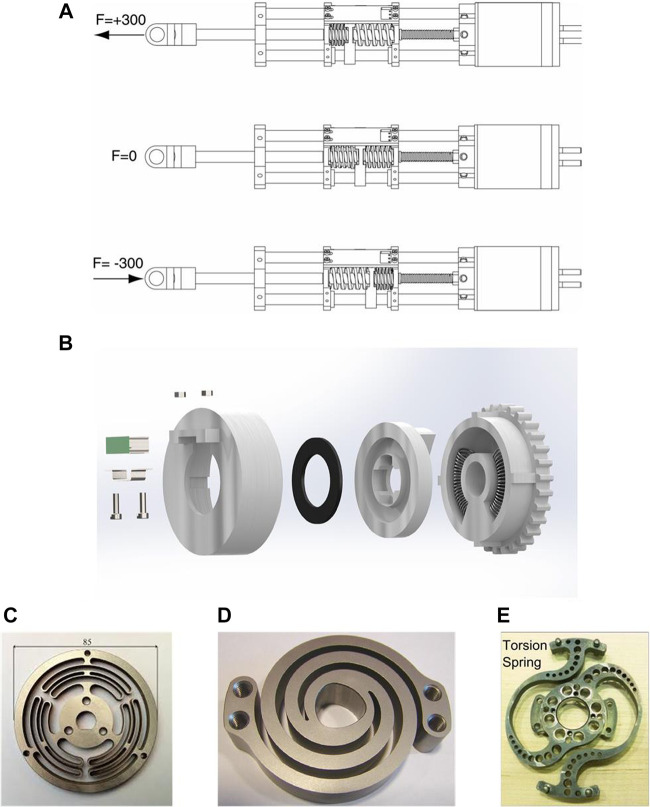
Different designs of the elastic element **(A)** series elastic actuator SEA23-23 from Yobotics (Pratt et al., 1997, 2002) **(B)** series elastic actuator from Serpens robot (Sanfilippo et al., 2019a) **(C)** a torsional elastic element of wearable robots for knee assistance (Carpino et al., 2012) **(D)** LOPES′ spring (Lagoda et al., 2010) **(E)** NASA Valkyrie’s spring (Paine et al., 2015; Radford et al., 2015).

Elastic elements play a very important role in elastic joints. They are responsible for absorbing unexpected collision and storing elastic energy. The elastic elements can be springs and can have different shapes (as shown in Figure 2). Springs in elastic joints can be classified into torsion spring and extension/compression spring. In (Pratt et al., 1997, 2002; for; Human and Cognition, 2020), a pair of extension/compression springs are used, as shown in Figure 2A. In (Sanfilippo et al., 2019a), two springs are bent to fit into a compact housing, as shown in Figure 2B. Beside conventional springs, there are some custom designs of the elastic element. Some examples are shown in Figure 2.

To amplify the torque and reduce the speed of a motor, gearing systems are adopted. Besides the common spur gears, some special types of gear are also utilised in elastic joints. Strain wave gearing, also known as harmonic gearing, can be applied, e.g., (Negrello et al., 2015; Wang et al., 2017; Sergi et al., 2012). This mechanism consists of three basic components: a wave generator, a flex spline, and a circular spline. The advantages of the harmonic gearing mechanism are no backlash, high compactness, lightweight, high gear ratios in a small volume, and coaxial input and output shafts. In the research of Meng (Wang et al.,2015; Wang et al., 2017), a nonlinear series elastic joint with variable stiffness is presented. There are two motors and a harmonic gearing mechanism in this design. One motor is connected to the wave generator, while the other motor is used to change the stiffness preset, and the link is attached to the flex spline. Planetary gearing system is also utilised in elastic joints, e.g., (Lee and Oh, 2016; Plooij et al., 2016). The advantages of the planetary gearing mechanism are compactness, high efficiency, low backlash, high torque density (i.e., high torque-to-mass ratio), and coaxial input and output shafts. In Lee and Oh (2016), good control performance is achieved while the size is still compact. The motor shaft is connected to the Sun gear, the torsional spring is connected to the ring gear, the load is attached to the carrier and the planet gears combine these three parts. One side of the spring and the motor is attached to a fixed base. In Plooij et al. (2016), a *BIdirectional Clutched Parallel Elastic Actuator* (BIC-PEA) is introduced. The mechanism contains a planetary differential, a torsion spring, and two brakes. It is stated that BIC-PEA design can save up to 65% of the energy consumption in some specific tasks.

## 3 Control Algorithm

In this section, a mathematical model of the considered elastic joint system is introduced. A novel control algorithm was previously designed by our research group and proposed in (Hua et al., 2019). This control algorithm is summarised in this section. For further details, the reader is referred to (Hua et al., 2019).

### 3.1 Mathematical Model

**FIGURE 3:**
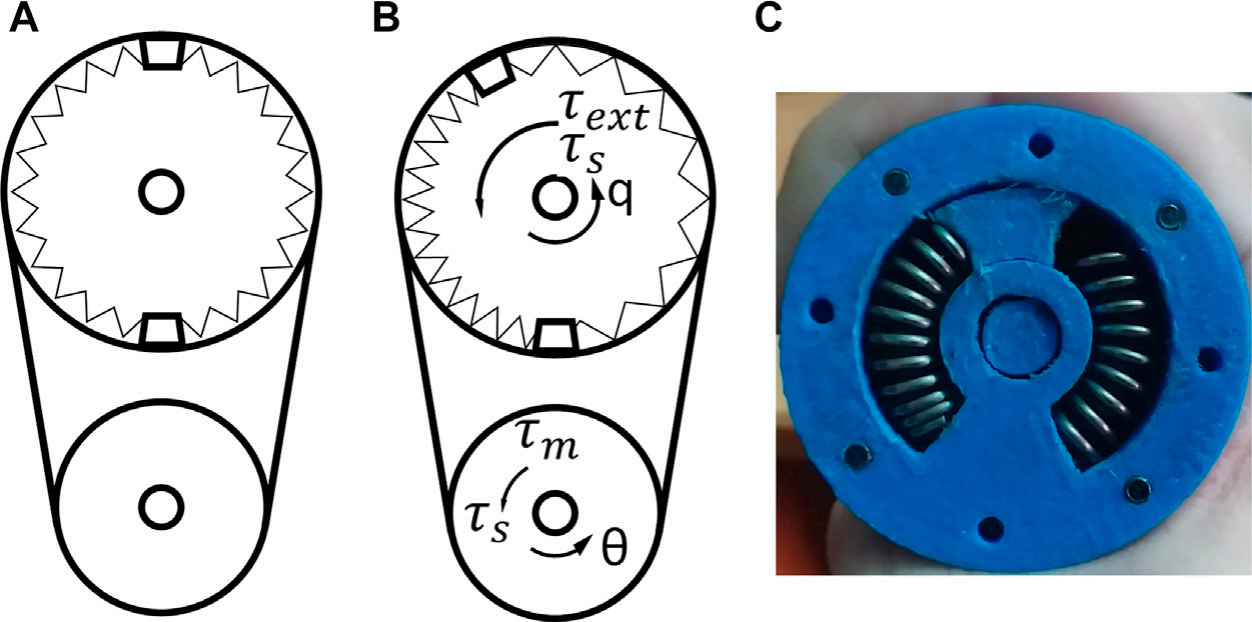
Elastic joint system **(A)** without external force/torque **(B)** compressed/tensed by external action **(C)** compression of the springs on the real prototype.

The schematic diagrams of the elastic actuator are illustrated in Figure 3. There are two gears with gear ratio $N=N_{l}/N_{m}$, where $N_{l}$ and $N_{m}$ are the number of teeth for the load and the motor gear, respectively. The torques shown in these diagrams are motor torque ($\tau_{m}$), spring reaction torque ($\tau_{s}$) and external torque ($\tau_{ext}$). Figure 3A illustrates the system when there is no external force/torque, while Figure 3B illustrates the system affected by an external action. The compression of the springs on the real prototype is also shown on Figure 3C.

The whole system is affected by disturbances from the motor ($d_{m}$) as well as from the load ($d_{l}$). These parameters represent external noise and noise caused by electromechanical systems (e.g., vibrations of the mechanical parts, voltage spike in electronic components, etc). By denoting the motor angular position as θ, the load angular position as *q*, the rotor inertia as $J_{m}$, the motor damping coefficient as $D_{m}$, the stiffness coefficient of the spring as $K_{s}$, the spring damping coefficient as $D_{s}$, the load inertia as $J_{l}$, and the load damping coefficient as $D_{l}$, the mathematical model of the elastic joint system is obtained:

$$d_{m}+\tau_{m}-N^{-1}\tau_{s}=J_{m}\ddot{\theta }+D_{m}\dot{\theta },$$ (1)

$$\tau_{s}=K_{s}\left(N^{-1}\theta -q\right)+D_{s}\left(N^{-1}\dot{\theta }-\dot{q}\right),$$ (2)

$$d_{l}+\tau_{s}+\tau_{ext}=J_{l}\ddot{q}+D_{l}\dot{q}.$$ (3)

Eq. 1 shows the relationship on the motor-side between the motor torque, the spring torque and the motor angular position. The spring torque, $\tau_{s}$, is obtained by Eq. 2. The interaction between the spring torque, the external torque and the load angular position is illustrated by Eq. 3.

### 3.2 Controller Design

**FIGURE 4:**
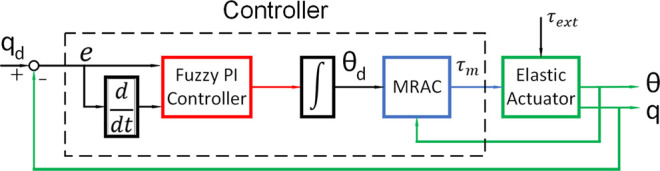
The proposed two-loop controller.

The proposed control algorithm diagram is presented in Figure 4. The objective of this work is to develop a controller that can track the desired load angular position when considering external disturbances on the load and uncertainties in system parameters. To achieve this objective, two separate types of controllers are utilised for the motor-side and the load-side, respectively. For the load-side, we propose using a Fuzzy PI Controller (FPIC) to reduce the effect of external disturbances on the load. The considered external disturbances are the forces/torques caused by undesired collisions when operating in unknown environments. The output of the FPIC is used as the desired angular position of the motor. For the motor-side, a Model Reference Adaptive Controller (MRAC) is used to cope with uncertainties in system parameters. The uncertain system parameters could be the inertia of the load or the stiffness of the spring. The idea is inspired to a previous research of (Losey et al., 2016). The advantage of combining the FPIC and the MRAC controllers is the possibility of achieving independence with respect to imprecise system parameters. In previous works (Losey et al., 2016), the motor angular position is prioritised more than the load position because the target is force control. The contribution of the paper is that a robust position control algorithm for the load position is proposed. In addition, a better spring model is given with the damping coefficient added.

#### 3.2.1 Fuzzy PI Controller

An FPIC is applied to the load-side to cope with the effect of external disturbances on the elastic joint system. A fuzzy controller is not based on a strict mathematical model and is widely used to solve problems under uncertain environments, with high nonlinearities (Al-Odienat and Al-Lawama, 2008). A fuzzy algorithm is a control method based on fuzzy logic, which uses “fuzzy inference rules” instead of “equations”. These fuzzy inference rules may come from experience of a human expert in controlling a specific object, or in other cases, from the understanding of dynamics and behaviour of the target plant. A fuzzy controller can be combined with a conventional PID controller to obtain a fuzzy PI, a fuzzy PD or a fuzzy PID controller. The fuzzy PI type controller is known to be more practical than fuzzy PD types because it is difficult for the fuzzy PD to remove steady state errors (Rao et al., 2010). The relationship between the input and the output in a conventional PI controller is expressed in the following equation:

$$u\left(t\right)=K_{p}e\left(t\right)+K_{i}{\int^{\text{​}}}_{0}^{t}e\left(t\right)dt,$$ (4)

where, Kp and Ki are scaling the coefficient of the feedback error, the change of feedback error and the change of control signal, respectively. In fuzzy control, the experience of the human operator is applied to establish fuzzy inference rules. However, the integral of the error is more difficult to be analysed than the change of the error. Therefore, the control signal u is differentiated so that the change of the error can be considered. Then, by differentiating Eq. 4, we obtain:

$$\dot{u}\left(t\right)=K_{p}\dot{e}\left(t\right)+K_{i}e\left(t\right).$$ (5)

A fuzzy PI controller can be obtained by combining Eq. 5 with the fuzzy controller. Inputs of the FPIC are the feedback error (*e*) and the change of this error ($\dot{e}$), while the output of the FPIC is the change of control signal ($\dot{u}$).

**FIGURE 5:**
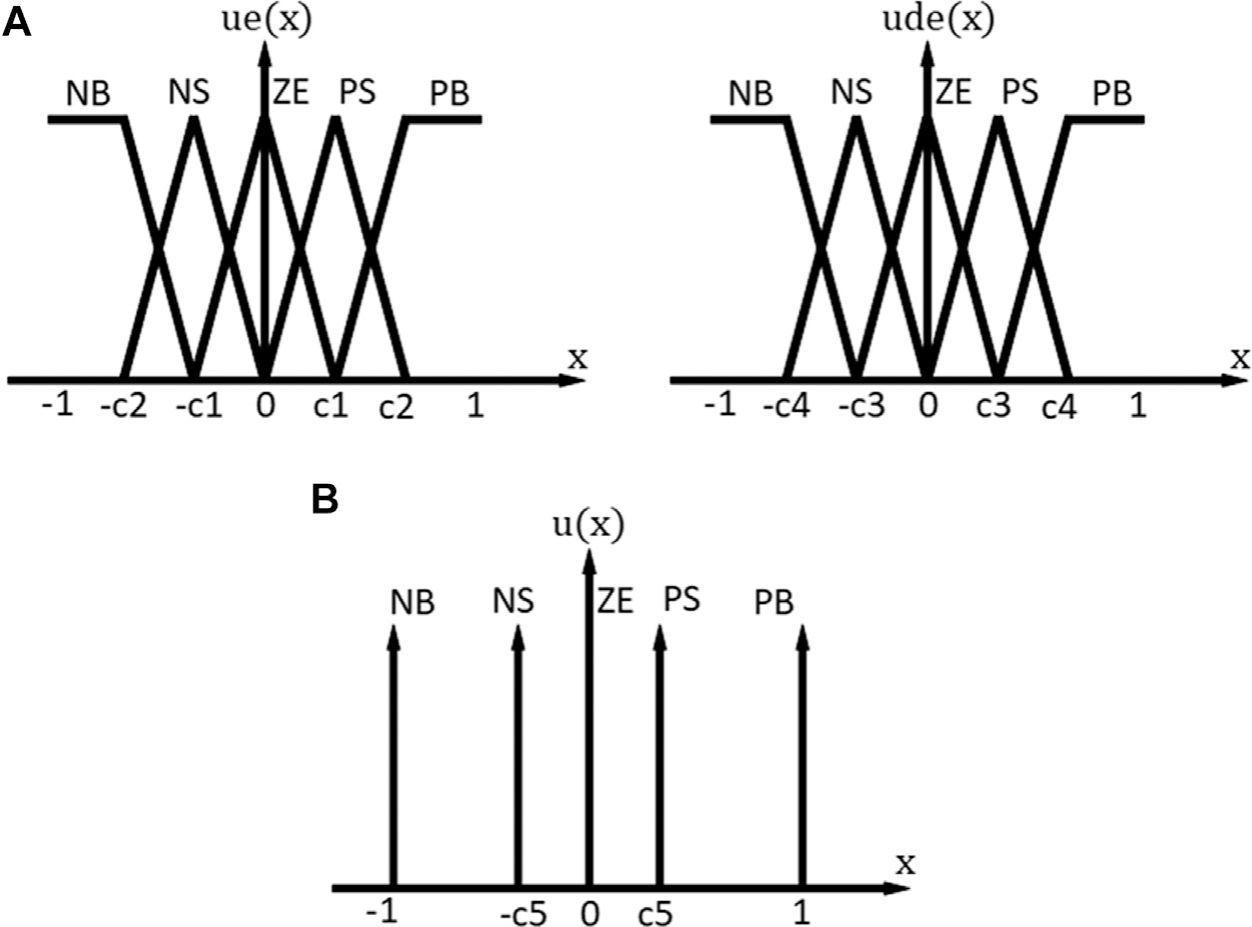
Fuzzy membership functions **(A)** input membership functions **(B)** output membership functions.

The membership functions for the input and the output are shown in Figure 5A,B, in which c1−c5 are parameters to be adjusted. These parameters are chosen by trial and error in the range of (0, 1). The narrower the membership functions are, the faster is the response at the expense of larger oscillations and overshoots. The membership functions for the error and the change of error are similar, including the rules base of the fuzzy model: Negative Big (NB), Negative Small (NS), Zero (ZE), Positive Small (PS), and Positive Big (PB). The membership functions for the change of the control signal are in singleton over the output, given NB, NS, ZE, PS, and PB. On the basis of the input and output membership functions, 25 fuzzy inference rules are established as shown in Table 1.

**TABLE 1:** Fuzzy inference rules.

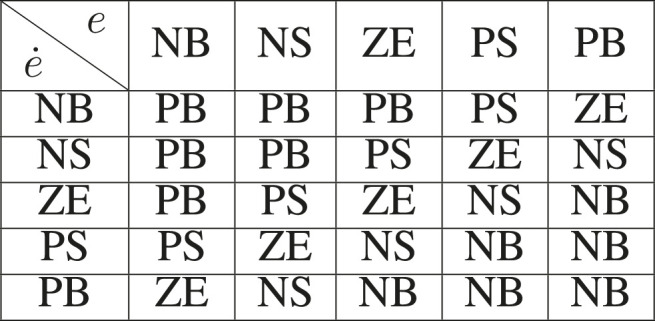

#### 3.2.2 Model Reference Adaptive Controller

Although there are various control algorithms available from the past literature (Pratt and Williamson, 1995; Talole et al., 2010; Losey et al., 2016; Penzlin et al., 2019), uncertainties in system parameters can lead to instability in many cases. Adaptive controllers are developed to overcome this problem. An MRAC is an important adaptive controller typology in which the desired response is expressed by a reference model. The adaptation law modifies the system parameters based on the difference between the output of the real system and the output of the reference model. In this article, Lyapunov’s stability theory is applied to design the adaptation law. Lyapunov’s stability criterion states that a system, $\dot{x}=f\left(x\right)$, with equilibrium point at x=0, is stable if there is a function, V(x), that satisfies the following conditions:

V(x)=0 if x=0, (6)

V(x)>0 if x≠0, (7)

$$\dot{V}\left(x\right)\leq 0\;for\;all\;x\neq 0.$$ (8)

Firstly, the control law is derived. The motor-side system equations can be rewritten by using Eqs. 1, 2 (ignoring the motor-side external disturbance $d_{m}$) as:

$$\begin{array}{l} {\dot{\theta }}_{m}=\left[\begin{array}{c} \dot{\theta }\\ \ddot{\theta } \end{array}\right]=\left[\begin{array}{cc} 0 & 1\\ \frac{-K_{s}N^{-2}}{J_{m}} & \frac{-D_{s}N^{-2}-D_{m}}{J_{m}} \end{array}\right]\left[\begin{array}{c} \theta \\ \dot{\theta } \end{array}\right]+\left[\begin{array}{c} 0\\ \frac{1}{J_{m}} \end{array}\right]\left(\tau_{m}+K_{s}N^{-1}q+D_{s}N^{-1}\dot{q}\right)\\ =A\theta_{m}+B\left(\tau_{m}+K_{s}N^{-1}q+D_{s}N^{-1}\dot{q}\right). \end{array}$$ (9)

In Eq. 9, the state matrix *A*, input matrix *B* and state vector $\theta_{m}$ of the system can be obtained as:

$$A=\left[\begin{array}{cc} 0 & 1\\ \frac{-K_{s}N^{-2}}{J_{m}} & \frac{-D_{s}N^{-2}-D_{m}}{J_{m}} \end{array}\right],B=\left[\begin{array}{c} 0\\ \frac{1}{J_{m}} \end{array}\right],\theta_{m}=\left[\begin{array}{c} \theta \\ \dot{\theta } \end{array}\right].$$ (10)

The motor-side system equations have the form of a second-order system, so the reference model is a second-order system model with the desired signal $\theta_{md}$, natural frequency $\omega_{n}$, and damping coefficient ξ:

$$\begin{array}{c} {\dot{\theta }}_{ref}=\left[\begin{array}{c} {\dot{\theta }}_{r}\\ {\overset{..}{\theta }}_{r} \end{array}\right]=\left[\begin{array}{cc} 0 & 1\\ -\omega_{n}^{2} & -2\xi \omega_{n} \end{array}\right]\left[\begin{array}{c} \theta_{r}\\ {\dot{\theta }}_{r} \end{array}\right]+\left[\begin{array}{c} 0\\ \omega_{n}^{2} \end{array}\right]\theta_{md}\\ =A_{r}\theta_{ref}+B_{r}\theta_{md}. \end{array}$$ (11)

In Eq. 11, the state matrix $A_{r}$, input matrix $B_{r}$ and state vector $\theta_{ref}$ of the reference model can be obtained as:

$$A_{r}=\left[\begin{array}{cc} 0 & 1\\ -\omega_{n}^{2} & -2\xi \omega_{n} \end{array}\right],\;\;B_{r}\;\;=\left[\begin{array}{c} 0\\ \omega_{n}^{2} \end{array}\right],\;\;\theta_{ref}=\left[\begin{array}{c} \theta_{r}\\ {\dot{\theta }}_{r} \end{array}\right].$$ (12)

A general control law for the system with state Eq. 9 is:

$$\tau_{m}=M\theta_{md}-L\theta_{m}-{\hat{K}}_{s}N^{-1}q-{\hat{D}}_{s}N^{-1}\dot{q}.$$ (13)

Parameters with hats (${\hat{D}}_{m}$, ${\hat{J}}_{m}$, ${\hat{K}}_{s}$, ${\hat{D}}_{s}$, ${\hat{D}}_{l}$, ${\hat{J}}_{l}$) are estimated parameters, while matrices *M* and *L* need to be determined. By substituting $\tau_{m}$ to system Eq. 9, we have:

$${\dot{\theta }}_{m}=\left(A-BL\right)\theta_{m}+BM\theta_{md}+B\left(\left(K_{s}-{\hat{K}}_{s}\right)N^{-1}q+\left(D_{s}-{\hat{D}}_{s}\right)N^{-1}\dot{q}\right).$$ (14)

If perfect estimation of parameters is obtained, we have ${\hat{K}}_{s}=K_{s}$ and ${\hat{D}}_{s}=D_{s}$. As the columns of matrices $A-A_{r}$ and $B_{r}$ are linear combinations of the vector *B*, there exists optimal matrices $M^{\text{*}}$ and $L^{\text{*}}$ of matrices *M* and *L* such that:

$$A-A_{r}=BL^{\text{*}},$$ (15)

$$B_{r}=BM^{\text{*}}.$$ (16)

Eqs. 15, 16 are called compatible conditions. If these conditions are satisfied and we have perfect estimation of parameters, then the controller 13 can yield perfect tracking of the reference model. From these equations, the controller optimal matrices $M^{\text{*}}$ and $L^{\text{*}}$ can be obtained as below:

$$M^{\text{*}}={\left(B^{T}B\right)}^{-1}B^{T}B_{r}=\omega_{n}^{2}J_{m},$$ (17)

$$\begin{array}{cc} & L^{\text{*}}={\left(B^{T}B\right)}^{-1}B^{T}\left(A-A_{r}\right)\\ & =\left[\begin{array}{cc} \left(-K_{s}N^{-2}+J_{m}\omega_{n}^{2}\right) & \left(-D_{s}N^{-2}-D_{m}+2J_{m}\xi \omega_{n}\right) \end{array}\right]. \end{array}$$ (18)

Based on the above Eqs. 17, 18 for the controller optimal matrices, the matrices *M* and *L* can be approximated as:

$$M={\left(B^{T}B\right)}^{-1}B^{T}B_{r}=\omega_{n}^{2}{\hat{J}}_{m},$$ (19)

$$\begin{array}{cc} & L={\left(B^{T}B\right)}^{-1}B^{T}\left(A-A_{r}\right)\\ & =\left[\begin{array}{cc} \left(-{\hat{K}}_{s}N^{-2}+{\hat{J}}_{m}\omega_{n}^{2}\right) & \left(-{\hat{D}}_{s}N^{-2}-{\hat{D}}_{m}+2{\hat{J}}_{m}\xi \omega_{n}\right) \end{array}\right]. \end{array}$$ (20)

Secondly, the error equation is determined. The feedback error, which is the difference between the output of the real system and the output of the reference model, is determined as:

$$e=\theta_{m}-\theta_{ref}.$$ (21)

The derivative of error is determined as:

$$\begin{array}{c} \dot{e}={\dot{\theta }}_{m}-{\dot{\theta }}_{ref}=\left(A-BL\right)\theta_{m}+BM\theta_{md}+B\left(\left(K_{s}-{\hat{K}}_{s}\right)N^{-1}q+\left(D_{s}-{\hat{D}}_{s}\right)N^{-1}\dot{q}\right)\\ -\left(A_{r}\theta_{ref}+B_{r}\theta_{md}\right)\\ =A_{r}e-A_{r}\theta_{m}+\left(A-BL\right)\theta_{m}+BM\theta_{md}+B\left(\left(K_{s}-{\hat{K}}_{s}\right)N^{-1}q+\left(D_{s}-{\hat{D}}_{s}\right)N^{-1}\dot{q}\right)-B_{r}\theta_{md}\\ =A_{r}e+\text{Ψ}\left(\text{Φ}-{\text{Φ}}^{\text{*}}\right), \end{array}$$ (22)

where, it should be noted that:

$$\begin{array}{cc} & \text{Ψ}=B\left[\begin{array}{c} -\theta_{m}^{T}\;\theta_{md}\;\left(-N^{-1}q\right)\;\left(-N^{-1}\dot{q}\right) \end{array}\right],\\ & \text{Φ}=\left[\begin{array}{c} L^{T}\\ M\\ {\hat{K}}_{s}\\ {\hat{D}}_{s} \end{array}\right],{\text{Φ}}^{\text{*}}=\left[\begin{array}{c} L^{\text{*}T}\\ M^{\text{*}}\\ K_{s}\\ D_{s} \end{array}\right]. \end{array}$$ (23)

Thirdly, the adaption law can be obtained by applying Lyapunov’s stability theory. A Lyapunov function is introduced as:

$$V=\frac{1}{2}\gamma e^{T}Pe+\frac{1}{2}{\left(\text{Φ}-{\text{Φ}}^{\text{*}}\right)}^{T}\left(\text{Φ}-{\text{Φ}}^{\text{*}}\right),$$ (24)

where, *P* is a symmetric positive definite matrix and γ is a learning rate. The function *V* is positive definite. The derivative of *V* is obtained as:

$$\begin{array}{c} \dot{V}=\frac{1}{2}\gamma {\dot{e}}^{T}Pe+\frac{1}{2}\gamma e^{T}P\dot{e}+{\left(\text{Φ}-{\text{Φ}}^{\text{*}}\right)}^{T}\dot{\text{Φ}}\\ =-\frac{1}{2}\gamma e^{T}Qe+{\left(\text{Φ}-{\text{Φ}}^{\text{*}}\right)}^{T}\left(\dot{\text{Φ}}+\gamma {\text{Ψ}}^{T}Pe\right), \end{array}$$ (25)

where, $A_{r}^{T}P+PA_{r}=-Q$ and *Q* is a symmetric positive definite matrix. The existence of matrix *Q* is demonstrated by using the Kalman-Yakubovich lemma (Åström and Wittenmark, 2013). If the adaption law is chosen to be:

$$\dot{\text{Φ}}=-\gamma {\text{Ψ}}^{T}Pe,$$ (26)

then the derivative of Lyapunov function $\dot{V}$ is negative definite with all e≠0, which means that the feedback error between the output of the real system and the reference model will go to zero when time goes to infinity.

In this article, due to their symmetry, the matrices *P* and *Q* are chosen as it follows:

$$P=\left[\begin{array}{cc} p_{1} & p_{2}\\ p_{2} & p_{3} \end{array}\right],\;\;\;Q=\left[\begin{array}{cc} q_{1} & 0\\ 0 & q_{2} \end{array}\right],$$ (27)

where, $q_{1}$ and $q_{2}$ will be tuned appropriately. From equation $A_{r}^{T}P+PA_{r}=-Q$, $p_{1}$, $p_{2}$ and $p_{3}$ can be obtained by using the following formulas:

$$p_{2}=\frac{q_{1}}{2\omega_{n}^{2}},p_{3}=\frac{2p_{2}+q_{2}}{4\xi \omega_{n}},p_{1}=2\xi \omega_{n}p_{2}+\omega_{n}^{2}p_{3}.$$ (28)

In real applications, discrete system equations are utilized instead of continuous ones. By approximately discretising the reference system Eq. 9, we obtain:

$$\left[\begin{array}{c} \theta_{r}\left(k+1\right)\\ {\dot{\theta }}_{r}\left(k+1\right) \end{array}\right]=\left[\begin{array}{cc} 1 & T\\ -T\omega_{n}^{2} & 1-2T\xi \omega_{n} \end{array}\right]\left[\begin{array}{c} \theta_{r}\left(k\right)\\ {\dot{\theta }}_{r}\left(k\right) \end{array}\right]+\left[\begin{array}{c} 0\\ T\omega_{n}^{2} \end{array}\right]\theta_{md}.$$ (29)

## 4 Physical Prototype

### 4.1 Mechanical Design

**FIGURE 6:**
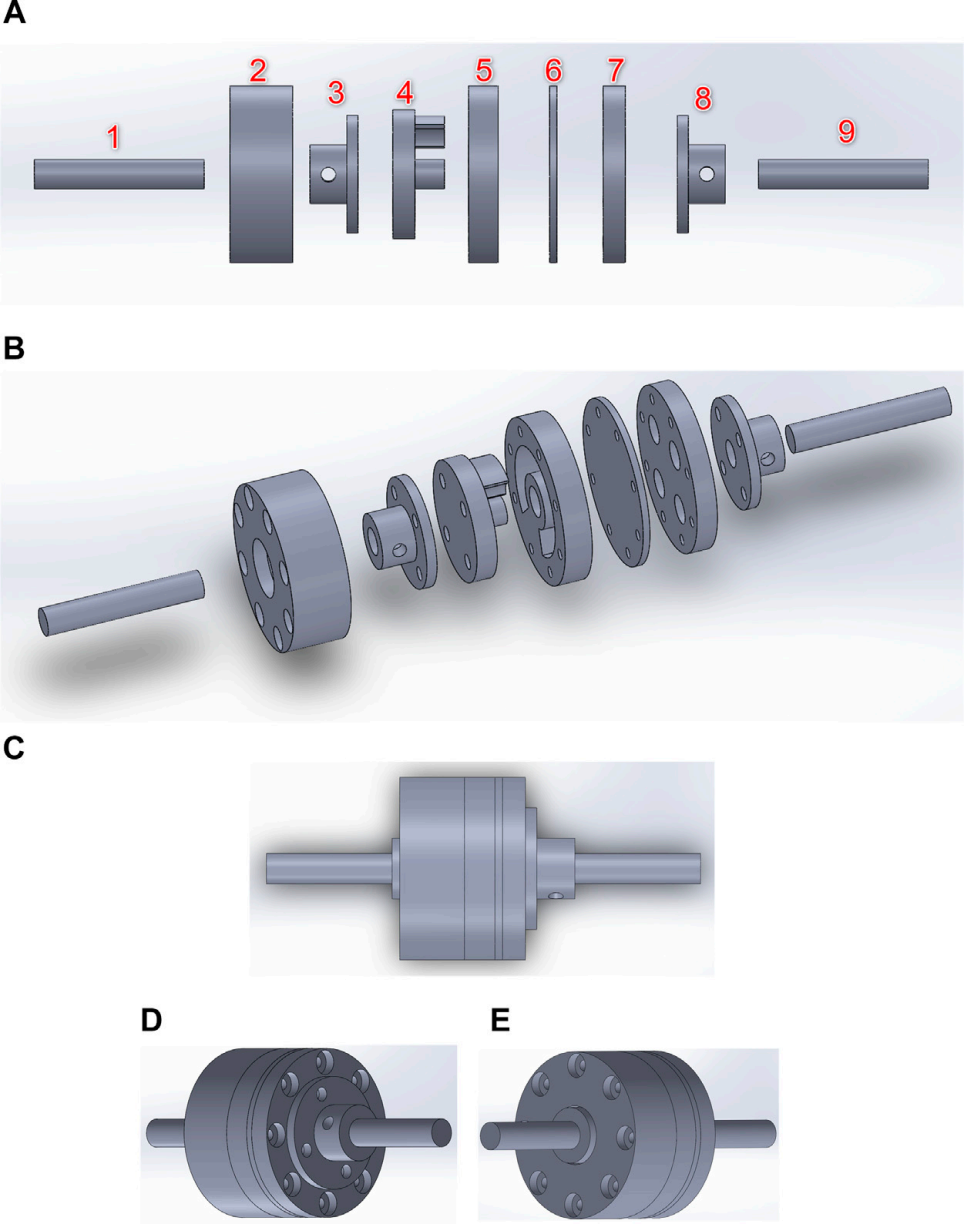
Mechanical design of the elastic joint **(A)** exploded view. Load side: (1) load side shaft, (2) cap, (3) 8 mm-shaft flange, (4) load side compliant joint. Motor side: (5) motor side driving joint, (6) cover, (7) flange extension, (8) 8 mm-shaft flange, (9) motor side shaft **(B)** 3D view of the elastic joint **(C)** assembled view of the design of the elastic joint **(D)** view from the motor side **(E)** view from the load side.

In this section, the mechanical design of the elastic joint and the robot arm are described. The mechanical components of the elastic joint are shown in Figure 6A. A 3D view of the same parts is illustrated in Figure 6B, while the assembled view of the actuator is illustrated in Figure 6C–E. The mechanism can be separated into load side and motor side. The motor side mechanism includes an 8 mm metal shaft, an 8 mm metal flange, a flange extension, a cover, and a driving joint (component 9, 8, 7, 6, 5 in Figure 6A, respectively). The load side mechanism includes an 8 mm metal shaft, a cap, an 8 mm metal flange, and a compliant joint (component 1, 2, 3, 4 in Figure 6A, respectively). The shafts are connected to the other parts by the flanges. The flange on the motor side is connected to the flange extension and the flange extension is connected to the cover and the motor side driving joint. The motor side driving joint has space to place two springs with 8 mm outer diameter. When the motor rotates, the springs are compressed. This will create an elastic force on the compliant joint. The load side shaft is connected to the joint through the flange. The shaft is kept in place by a cap. In our work, two extension/compression springs are chosen because they form a symmetrical configuration. In addition, the extension/compression type of spring is used instead of torsion spring because it is more durable and can bear a larger force.

**FIGURE 7:**
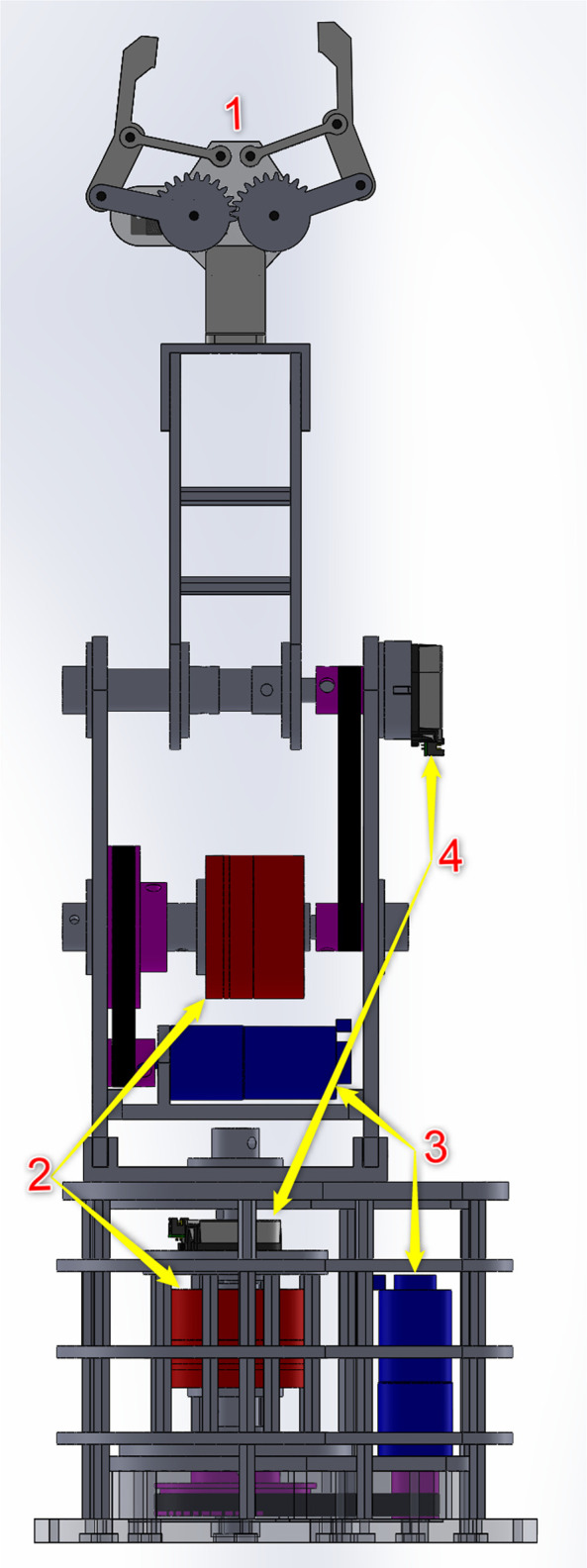
Mechanical design of the 2-DOF robot arm with elastic joints: (1) Gripper, (2) elastic joints, (3) GA25 DC motors, (4) AMT20 encoders.

Based on the newly introduced actuator, the design of the 2-DOF elastic robot arm is shown in Figure 7. The joints of the robot arm are revolute joints. The motor and the elastic joints are connected by pulleys and belts. The transmission ratio from the motor to the actuator is 4:1 (the pulley of the motor has 20 teeth and the pulley of the actuator has 80 teeth).

**FIGURE 8:**
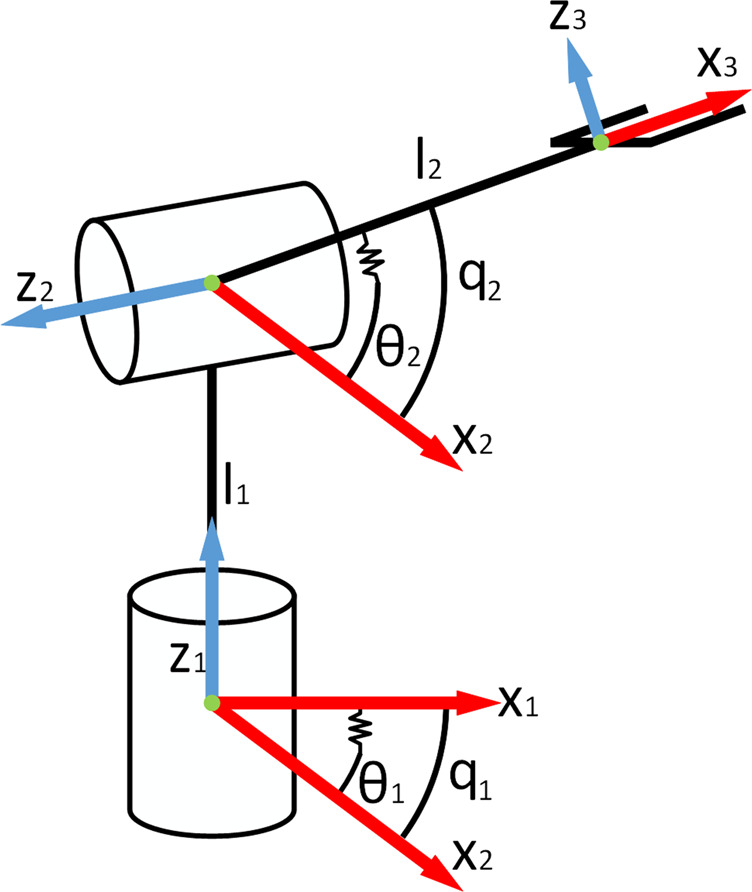
Diagram of the elastic robot arm.

The simplified model of the robot arm is shown in Figure 8. The Denavit-Hartenberg table is shown in Table 2. The angles are in radian and the lengths are in meter. From this table, the corresponding transformation matrices are formed, as shown in Eq. 30.

$$T_{1}^{2}=\left[\begin{array}{cccc} cos\left(q_{1}\right) & 0 & sin\left(q_{1}\right) & 0\\ sin\left(q_{1}\right) & 0 & -cos\left(q_{1}\right) & 0\\ 0 & 1 & 0 & l_{1}\\ 0 & 0 & 0 & 1 \end{array}\right],\;\;\;T_{2}^{3}=\left[\begin{array}{cccc} cos\left(q_{2}\right) & 0 & -sin\left(q_{2}\right) & l_{2}cos\left(q_{2}\right)\\ sin\left(q_{2}\right) & 0 & cos\left(q_{2}\right) & l_{2}sin\left(q_{2}\right)\\ 0 & -1 & 0 & 0\\ 0 & 0 & 0 & 1 \end{array}\right].$$ (30)

**TABLE 2:** Denavit-Hartenberg parameters.

	d	Θ	a	α
Joint 1	$l_{1}$	$q_{1}$	0	π/2
Joint 2	0	$q_{2}$	$l_{2}$	−π/2

### 4.2 Hardware Design

In this section, the hardware used in the robot arm is presented. The motor used in the robot arm is the GA25 DC motor (XYTmotor, 2020). It is a 12 V DC motor with a 4 mm shaft and a two channels encoder attached. The gearbox transmission ratio is chosen as 217:1. The encoder for the load side is the AMT-20 encoder (devices, 2020) from CUI Devices. It has incremental and absolute encoders in a compact package. The incremental encoder has three channels (A, B, and Z). The absolute encoder has 12 bits of absolute position information with Serial Peripheral Interface (SPI) communication. The microcontroller used is the STM32F446RE on the STM32 Nucleo board (Stmicroelectronics, 2020).

### 4.3 Software Design and Architecture

**FIGURE 9:**
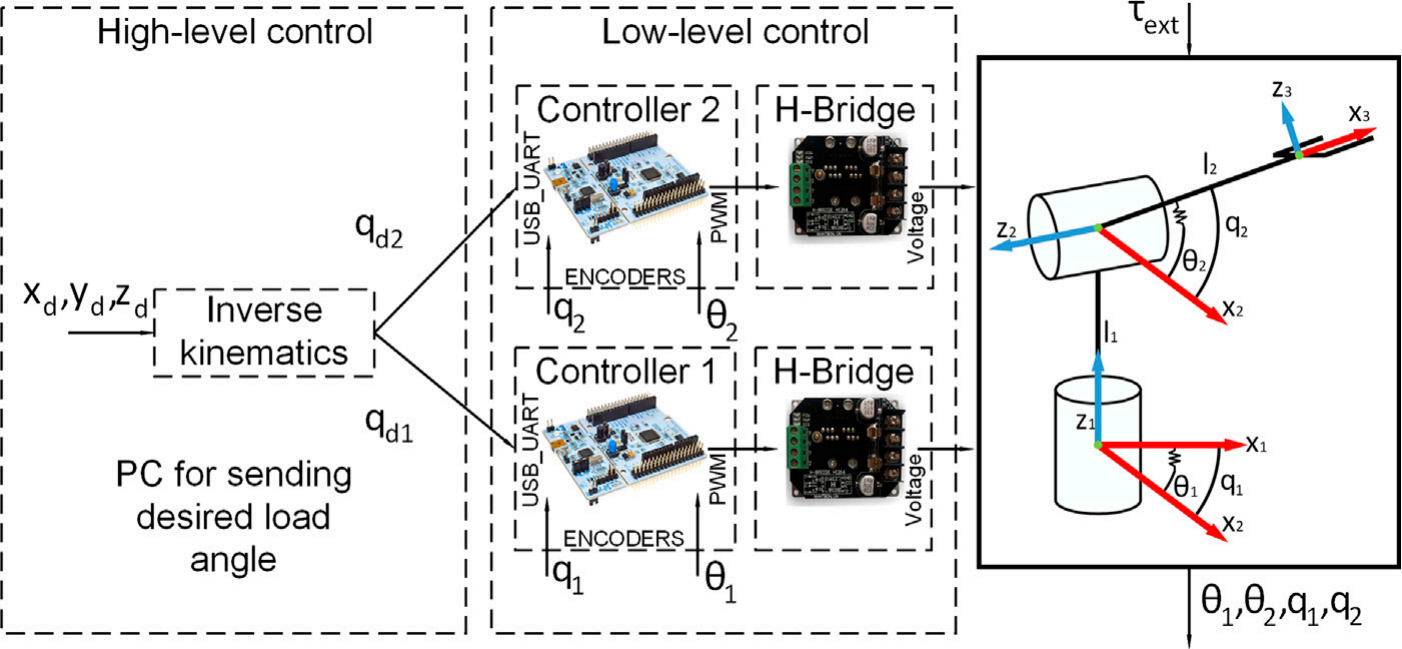
Software design.

The software design is described in this section. The concept of modularity is applied to the control architecture. Each joint is independent, being controlled by a self-reliant slave controller, which directly communicates with the master controller running on a personal computer (PC). Each slave controller communicates with the PC through a Universal Serial Bus (USB) port on the board. Figure 9 shows the diagram of the control architecture. Each STM32 board has timer peripherals, which are used to capture the encoder signals. The timers are also configured to be Pulse width modulation (PWM) output to control the motors through H-bridges (nhatbon, 2020). The control/update rate of the controller is 100 Hz (10 milliseconds). The presented framework is a multi-layer architecture that includes the following layers: High-level control layer: it considers the control of the overall manipulator, it includes kinematics according to the corresponding DOFs. Low-level control layer: it considers the control of the single joints. This makes it possible to use the proposed approach for robotic arms with different DOFs.

## 5 Simulation Results

In this section, simulations are outlined with the aim of validating the proposed control algorithm for the considered elastic joint. The simulations are conducted on (MATLAB, 2018). This software is chosen because of its ability to quickly constructing simulation models and validating control schemes. The responses of the elastic joint with step and sinusoidal wave inputs are presented. The effect of external torque and disturbances on the whole elastic system is also illustrated. The system parameters used in the simulation are shown in Table 3.

**TABLE 3:** System parameters.

Parameter	Value	Parameter	Value
Gear ratio(N)	1	Spring stiffness ($K_{s}$)	3
Load damping coefficient ($D_{l}$)	0.006	Motor damping coefficient ($D_{m}$)	0.06
Load inertia ($J_{l}$)	0.065	Motor inertia ($J_{m}$)	0.1
Spring damping coefficient ($D_{s}$)	0.6	Sampling rate (T)	0.001

### 5.1 Response of the Load-Side System With Step and Sine Desired Signals Without External Torque

**FIGURE 10:**
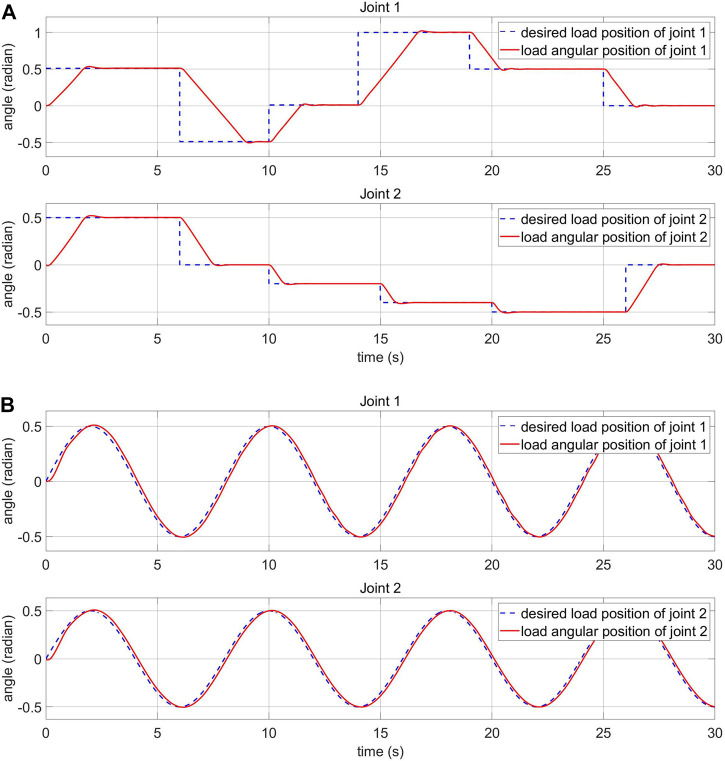
Load-side response of the FPIC-MRAC **(A)** step input **(B)** sine input.

The response of the load-side system in normal condition is illustrated in this section. Step and sine responses are shown in Figure 10. In these figures, blue dashed lines are desired inputs and red solid lines are load angular positions. The unstable stage at the beginning of the simulation is the learning phase of the MRAC. The results show that the proposed controller has relatively fast response and small error.

### 5.2 Response of the Motor-Side System With Step and Sine Desired Signals Without External Torque

**FIGURE 11:**
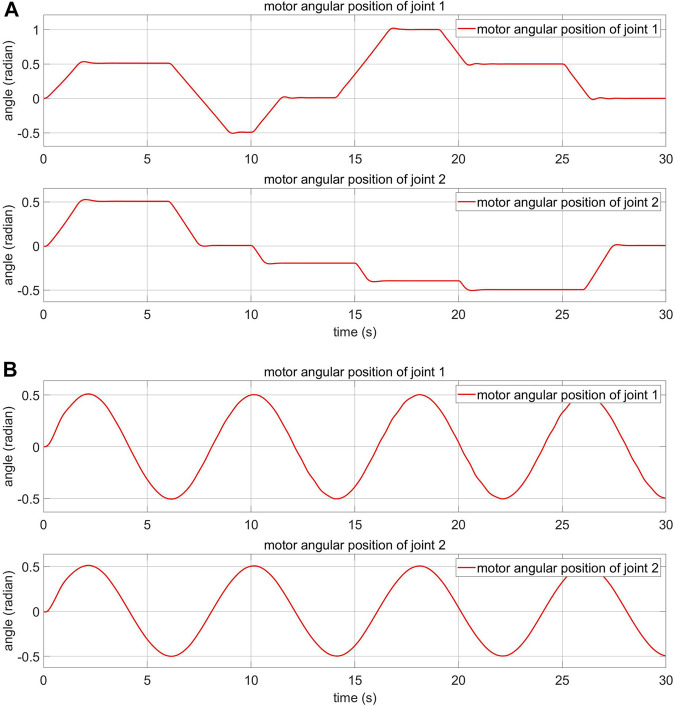
Motor-side response of the FPIC-MRAC **(A)** step input **(B)** sine input.

The response of the motor-side system in normal condition is presented in this section. Step and sine responses are shown in Figure 11A,B , respectively. The reference model and the MRAC parameters are shown in Table 4tbl4. The system parameters are modified based on the adaption law (Eq. 26) to reduce the error between reference model and real motor response. The adaption law is designed using Lyapunov’s approach. This has an advantage: arbitrary large values of the learning rate coefficient γ can be used (Åström and Wittenmark, 2013).

The damping coefficient ξ of the reference model is chosen to be critically damped (ξ=1) because there are no oscillations or overshoots in this configuration and the system returns to equilibrium in minimum time. However, Figure 11A shows that there is a small overshoot in the reference model graph. This is caused by the integrator in the FPIC, not by the reference model in the MRAC, and could be reduced by appropriately adjusting the $K_{p}$ coefficient. The rising time of the reference model is calculated by using the approximation formula: $\frac{5.83392}{\omega_{n}}$.

### 5.3 Response of the Load and Motor-Side System With External Torque to the Step Desired Signal

**FIGURE 12:**
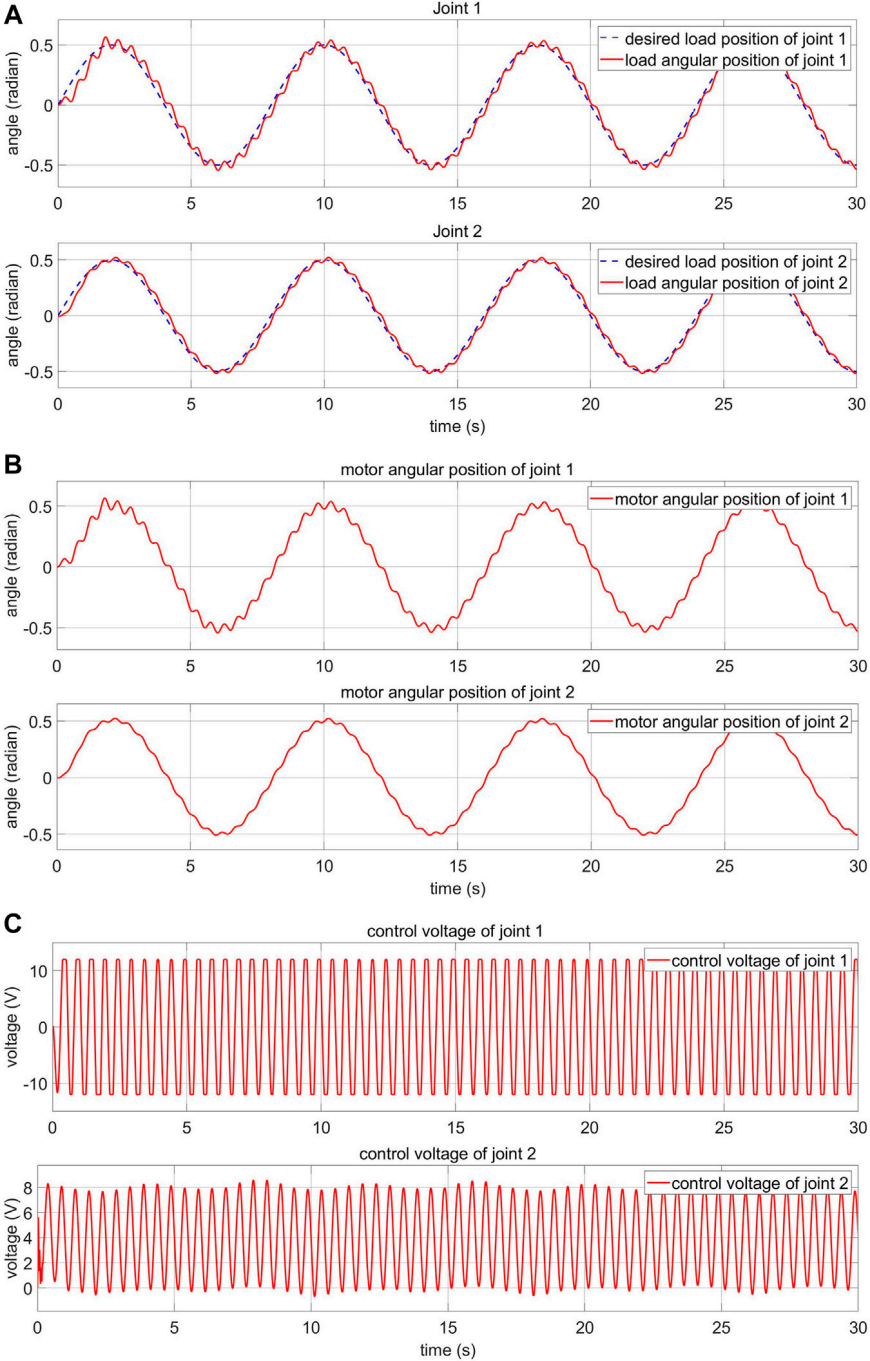
Response of the FPIC-MRAC of the robot arm with external disturbance **(A)** load-side response **(B)** motor-side response **(C)** motor control voltage.

In this section, the effect of the disturbance on the elastic joint and the efficiency of the proposed algorithm is demonstrated. The disturbance signal affecting the load side is a sine wave signal with an amplitude of five radian and a cycle time of 0.5 s. This signal is applied to each joint. This is equivalent to the vibrations often occurring in elastic joints. The sine response of the load-side system and the motor-side system is shown in Figure 12A,B, respectively. The motor control voltage is shown in Figure 12C. In the proposed two-loop controller, the FPIC is adopted on the load side to reduce the influence of external disturbances and the MRAC is adopted on the motor side to deal with uncertainties. When there is an external torque, the desired motor angular position is adjusted by the FPIC, so that the error between the desired load angular position and the real load angular position goes to zero. As shown in Figure 12B, the motor angular position changes when the external torque appears and the influence of this external torque is eliminated.

**TABLE 4:** Reference model and MRAC parameters.

Parameter	Value	Parameter	Value
Damping coefficient (ξ)	1	$p_{1}$	7
Natural frequency ($\omega_{n}$)	5	$p_{2}$	0.06
$q_{1}$	3	$p_{3}$	0.256
$q_{2}$	5	Learning rate coefficient (γ)	0.995

**FIGURE 13:**
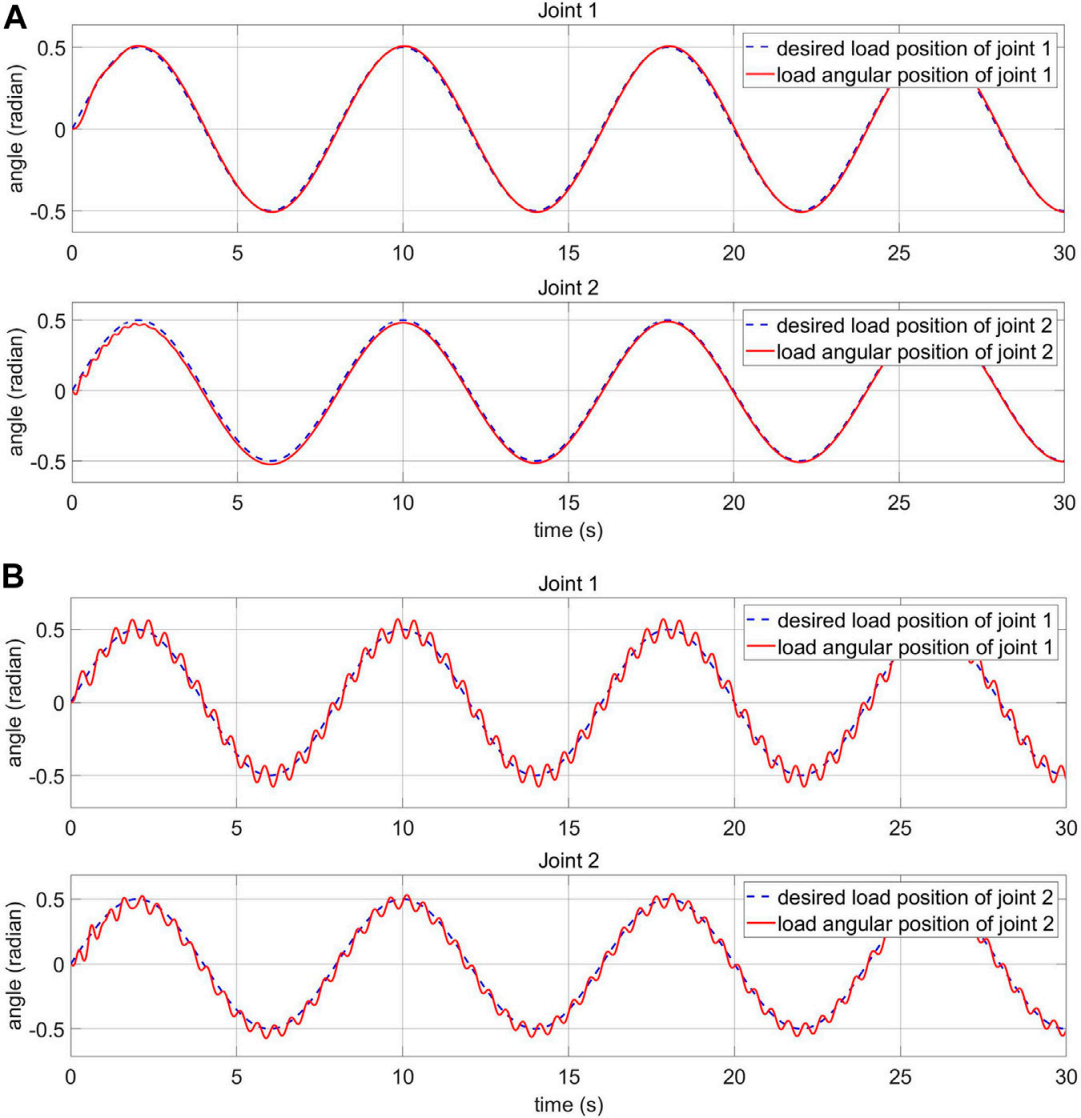
Load-side response of the PID controller of the robot arm **(A)** in normal condition **(B)** with external disturbance.

To show more evidently the effectiveness of the proposed control algorithm, a standard PID controller is applied and its results are compared. As shown in Figure 13A,B, the responses of the PID controller and the FPIC-MRAC controller are similar in normal condition. However, as shown in Figure 13B and Figure 12A, the response of the FPIC-MRAC controller is significantly better than the PID controller in the presence of external disturbance.

### 5.4 Response of the Load and Motor-Side System With Human-Machine Interaction

**FIGURE 14:**
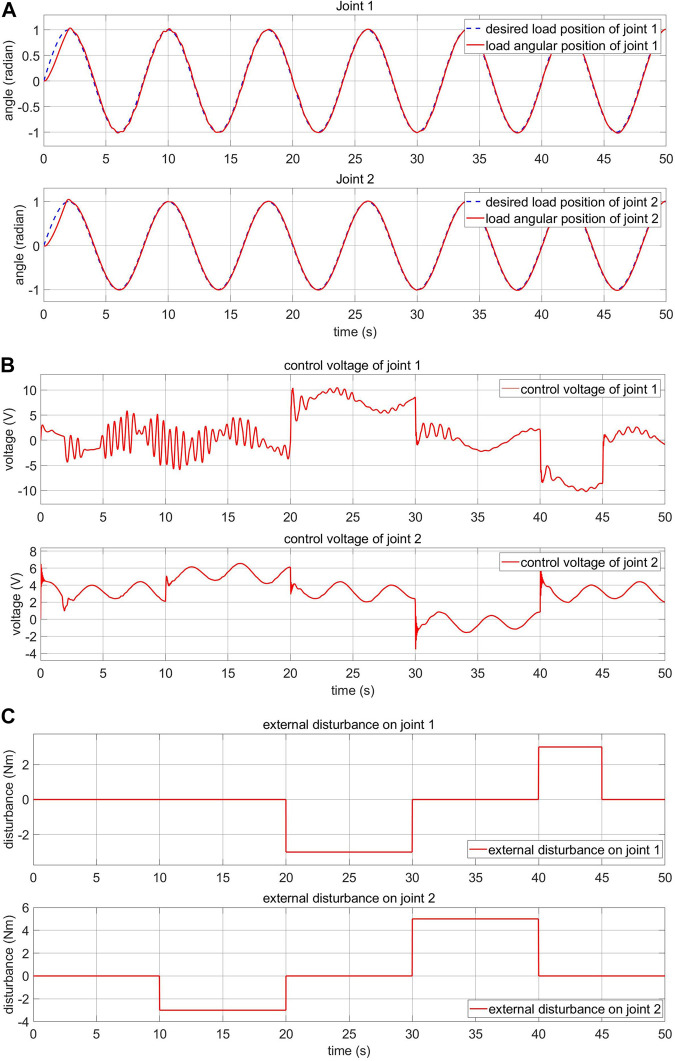
Load-side response and control voltage of the FPIC-MRAC with human-machine interaction **(A)** load-side response **(B)** control voltage **(C)** external disturbance.

The impact of human-machine interaction on the elastic joints is presented in this section. The load side response and the control voltage are shown in Figure 14. The simulated human-machine interaction of joint 1 appears from the second 20 to 30 and from 40 to 45. The simulated human-machine interaction of joint 2 appears from the second 10 to 20 and from 30 to 40. The collected data outline that despite being affected by the disturbance, the controller can adjust the voltage to keep the system stable.

## 6 Experiments

In this section, experiments are conducted to verify the effectiveness of the control algorithm on the mechanical prototype of the robot arm. The experiments consist of joints’ response and trajectory tracking. It should be noted that, in the simulation, the elastic joints are controlled directly by changing the voltage, as shown in Figure 12C. However, in the experiments, the elastic joints are controlled by torque produced from the motor which is proportional to the current injected by the H-bridge. This current is dependent on the applied voltage, which is controlled by adjusting the duty cycle of the PWM pulse.

### 6.1 Experiment in Normal Condition

In this subsection, experiment results related to the control of the real 2-DOF robot arm with elastic joints in the normal condition are presented. The normal condition is when the robot arm is not disturbed by the external torque. The joint angle response and control voltage are demonstrated. There are two input signal types: sinusoidal and square waveforms. The sinusoidal waveform input has an amplitude of 0.5 rad and a cycle time of 8 s. The square waveform input has an amplitude of 0.5 rad and a cycle time of 8 s. The data is collected within 50 s.

**FIGURE 15:**
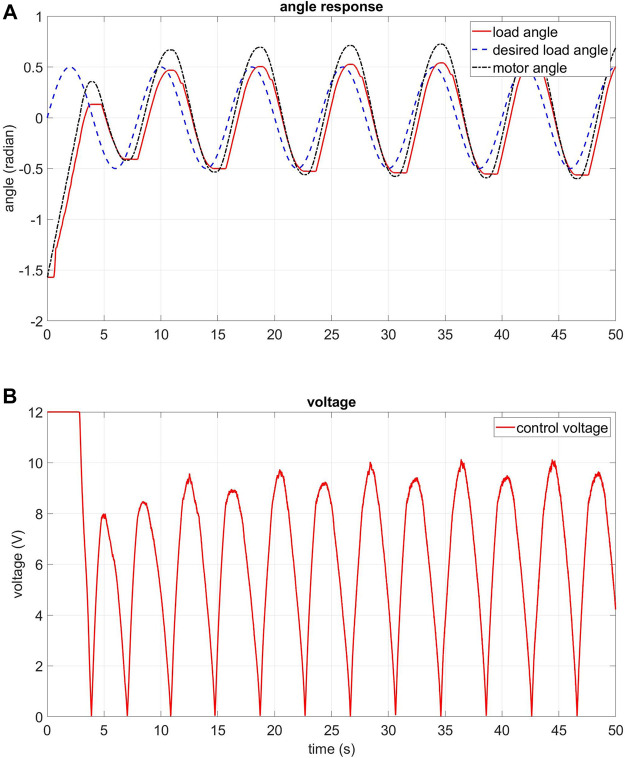
Experiment: angle response and control voltage of the FPIC-MRAC of joint 1 in normal condition, sine waveform input **(A)** angle response of joint 1 **(B)** control voltage of joint 1.

Figure 15; Supplement Figure S1 show the angle response and the control voltage of joint 1 and joint 2, respectively, in normal condition with the sinusoidal waveform input. The figures demonstrate that the decentralised control algorithm works as expected. As shown in Figure 15A, the absolute value of the positive peak of the motor angle is larger than the negative peak. This is due to a small manufacturing defect in the actuator: the spring on one side is stiffer than the spring on the other side. Despite this fact, it is clear that the controller still tracks the desired load angle.

The angle response and the control voltage of joint 1 and joint 2 are shown in Supplement Figures S2, S3, respectively, in normal condition with the square waveform input. These figures demonstrate that the decentralised control algorithm also works well in this situation. The error decreases to smaller than 10° after just a few seconds.

### 6.2 Experiment When There Is Unexpected Torque

In this subsection, the experiment results of the real 2-DOF robot arm with elastic joints in presence of disturbances are presented. The joint angle response and control voltage are demonstrated. The sinusoidal waveform input has an amplitude of 0.5 rad and a cycle time of 8 s. The data is collected within 50 s. The external disturbance is created by randomly holding the robot arm by hand while it is operating. This imitates the unexpected collision with a human operator while coexisting in a shared work-space.The angle response and the control voltage of joint 1 and joint 2 in disturbance condition are shown in Supplement Figures S4, S5, respectively. These figures demonstrate that the decentralised control algorithm can stabilise the system when there is unexpected torque. In addition, if the absolute value of the deviation between the motor angle and the load angle is larger than 30° (0.523,599 radians), the motor will stop. This value can be tuned appropriately, which means that safety can be guaranteed.

### 6.3 Trajectory Tracking Experiment in Normal Condition

In this subsection, trajectory tracking experiment results of the elastic robot arm in the normal condition are demonstrated. A sequence of images of the experiment is illustrated in Supplement Figure S6. The initial position is when $q_{1}=0,q_{2}=\frac{3\pi }{4}$ and the end position is when $q_{1}=\pi ,q_{2}=\frac{7\pi }{6}$. The desired trajectory and the response of the robot arm are shown in Supplement Figure S7A. It is clear that the end effector of the robot can be following the desired trajectory. In details, Supplement Figure S7B,C, show the responses of joints 1 and 2, respectively. The tracking error is relatively small.

### 6.4 Trajectory Tracking Experiment When There Is Unexpected Torque

In this subsection, trajectory tracking experimental results of the elastic robot arm when there is an unexpected torque are demonstrated. A sequence of images from the experiment is shown in Supplement Figure S8. The initial position is when $q_{1}=0,q_{2}=\frac{3\pi }{4}$ and the end position is when $q_{1}=\pi ,q_{2}=\frac{7\pi }{6}$. As shown in the third image of the sequence (Supplement Figure S8), the unexpected torque is produced by the collision with a human hand. The desired trajectory and the response of the robot arm are shown in Supplement Figure S9A. Obviously, there is a section where the end effector of the robot arm deviates from the desired trajectory. It corresponds to the period when the arm is affected by undesired torque. Supplement Figure S9B,C illustrate the responses of joints 1 and 2, respectively. As shown in these figures, when the collision occurred from second 7 to second 12, the robot arm tries to hold its position to mitigate the damage. After that, when the robot arm is released, it gradually converges to the end position.

## 7 Conclusion

A newly designed elastic joint was presented in this work based on our previuous design, which was previously introduced in Sanfilippo et al. (2019a), Sanfilippo et al. (2019b). Based on the developed elastic joint, a 2-DOF robot arm with elastic behaviour for safe human-robot interaction was also presented. The mechanical prototype can be 3D-printed by using Fused Deposition Modelling (FDM) manufacturing technology, thus making the rapid-prototyping process very economical and fast. Moreover, a position control algorithm was introduced to control each joint of the arm. The control algorithm is based on a 2-loops control mechanism. In particular, the inner control loop is designed as a model reference adaptive controller (MRAC) to deal with uncertainties in the system parameters, while the outer control loop utilises a fuzzy proportional-integral controller (FPIC) to reduce the effect of external disturbances on the load. Preliminary simulations were carried out in *Matlab* to validate the potential of the proposed control algorithm. Successively, the effectiveness of the controller was also proven by conducting experiments with the mechanical prototype.

As future work, the possibility of replacing the DC motors with brushless DC (BLDC) motors will be considered. Due to having high torque density, BLDC motors will make it possible to achieve a more compact actuation system. In addition, as shown in Figure 9, the proposed controller has a decentralised design. This means that each joint has its own separated controller, both in hardware and software. Therefore, the number of DOFs can be expanded and tested easily in the future. Furthermore, the stability analysis at the system level will also be considered.

Moreover, the possibility of realising a digital-twin of the presented robot will be considered to enable researchers for designing and prototyping different control algorithms. In this perspective, the integration with an open-source software framework will be investigated. To achieve this, there are different robotic frameworks and middleware available in recent years (Tsardoulias and Mitkas, 2017). However, the Robot Operating System (ROS) (Quigley et al., 2009) has emerged as a *de facto* standard for robot software architecture in the research community. In conjunction with ROS, Gazebo 3D simulator (Koenig and Howard, 2004) can be adopted to accurately and efficiently simulate robots in complex indoor and outdoor environments. Therefore, these tools will be considered to realise a digital-twin of the presented robot.

In the future, the design of reliable low-level control algorithms for the proposed elastic joints could be also relevant for other robotic systems, such as snake like robots similar to *Serpens* (Sanfilippo et al., 2019a), which is developed by our research group. This technology may be essential to enable the achievement of *perception-driven obstacle-aided locomotion* (POAL) (Sanfilippo et al., 2016, 2017, 2018).

## Data Availability

The raw data supporting the conclusion of this article will be made available by the authors, without undue reservation.
