# Supplementary figures and images for: Modelling and Control of a 2-DOF Robot Arm with Elastic Joints for Safe Human-Robot Interaction
Source: Front Robot AI. 2021 Aug 18;8:679304. doi: 10.3389/frobt.2021.679304 (PMC8416520; doi:10.3389/frobt.2021.679304)

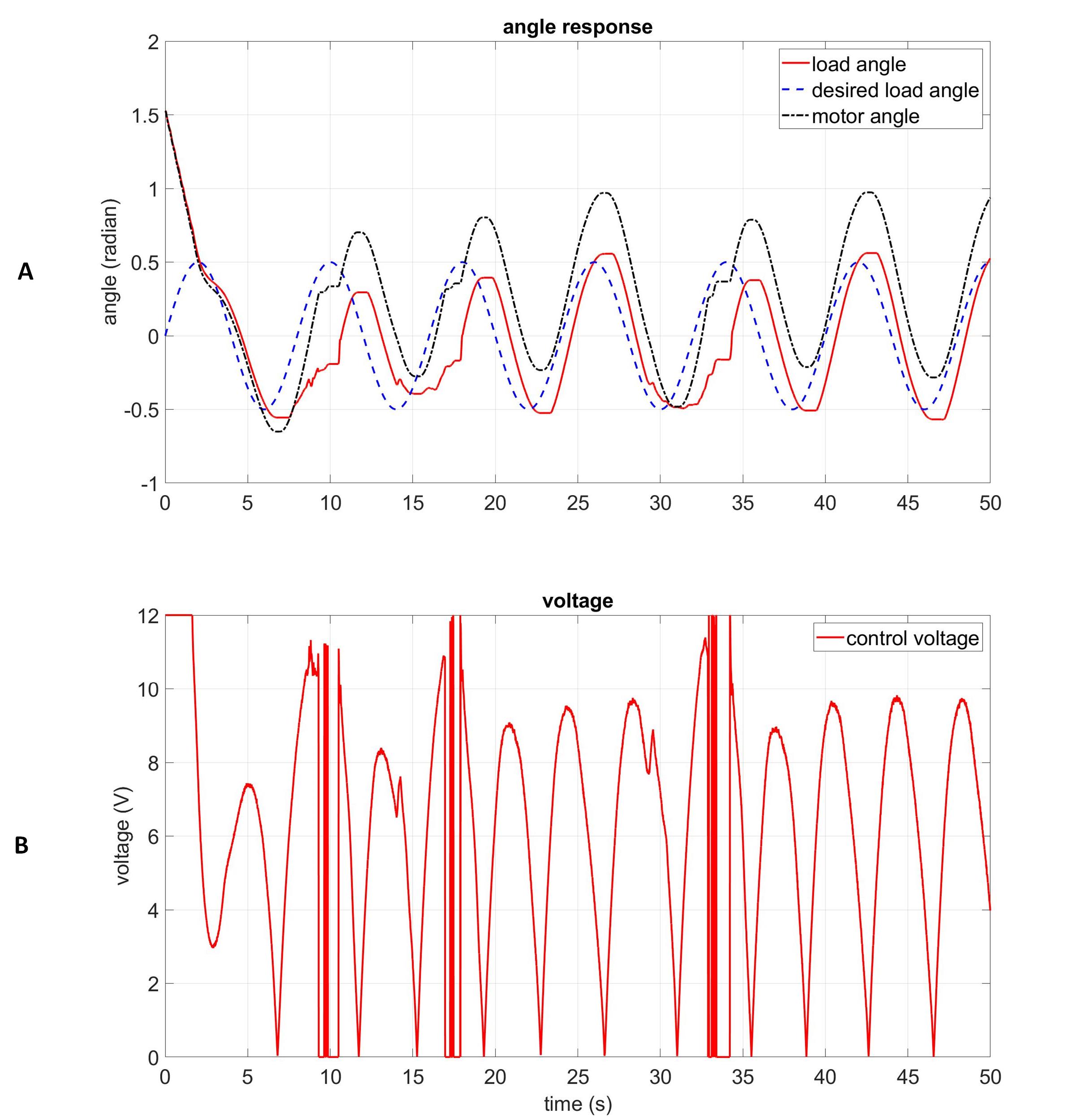

Supplement: Supplementary file 1 [file Image5.jpg]

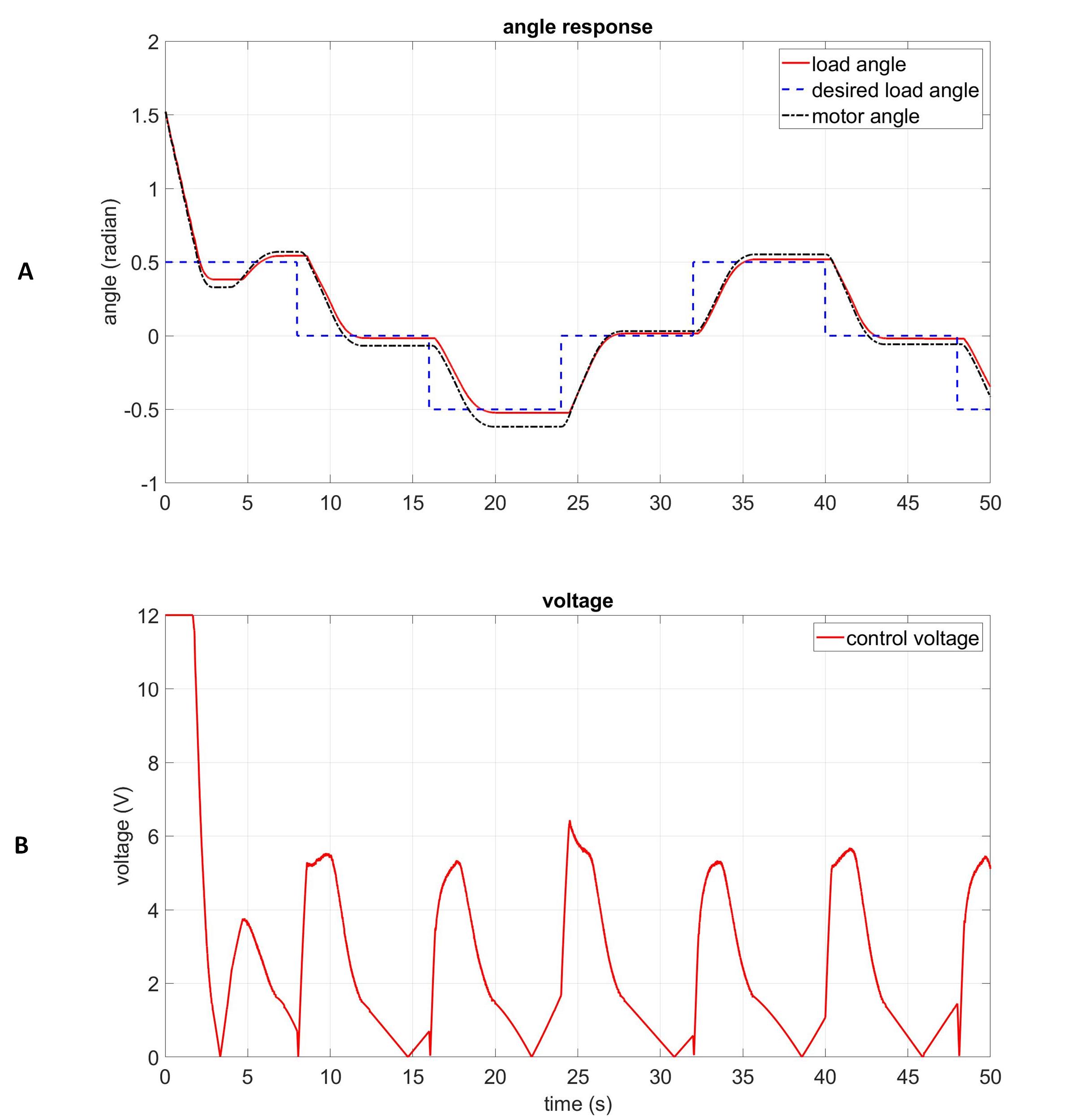

Supplement: Supplementary file 2 [file Image3.jpg]

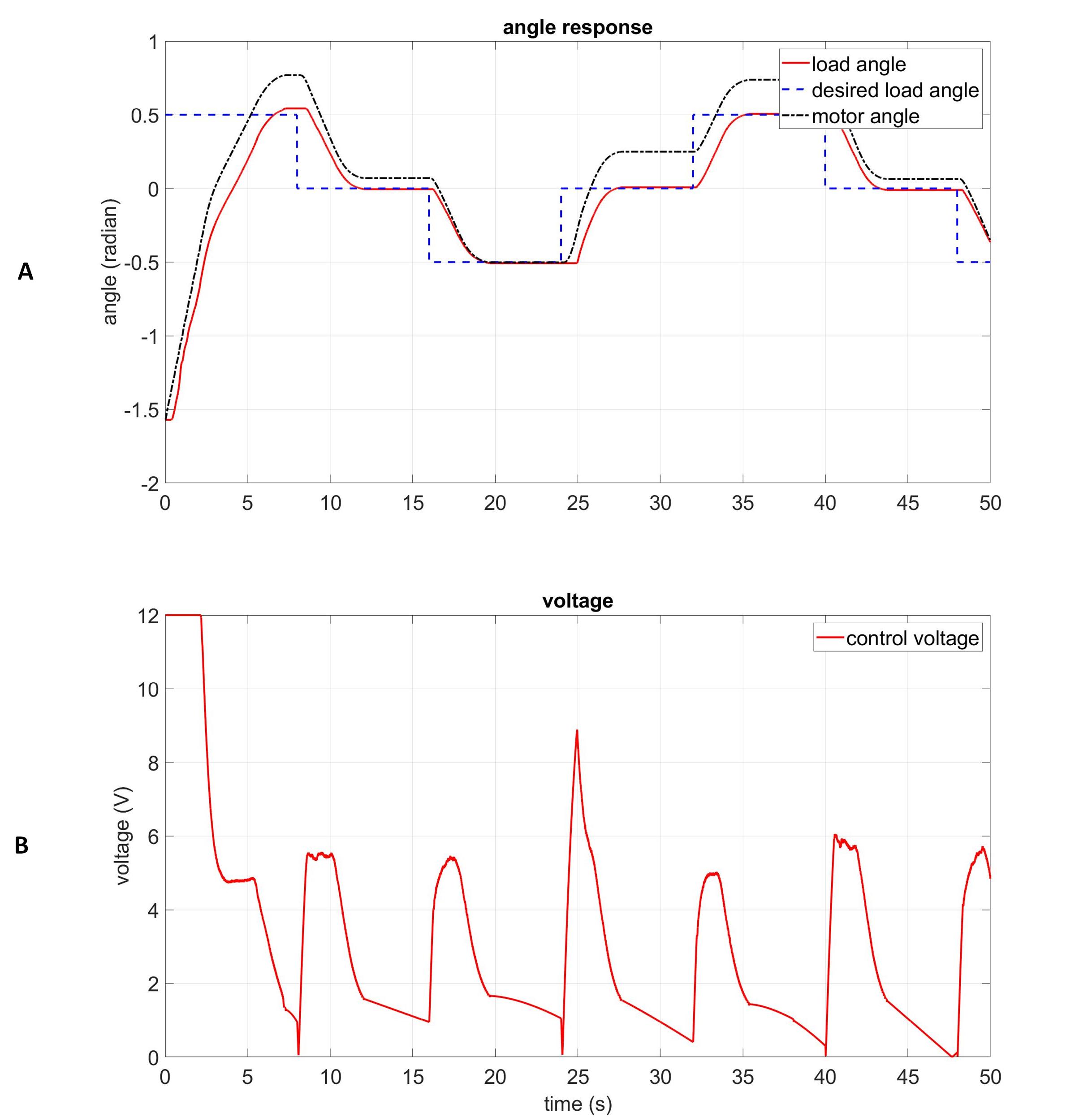

Supplement: Supplementary file 3 [file Image2.jpg]

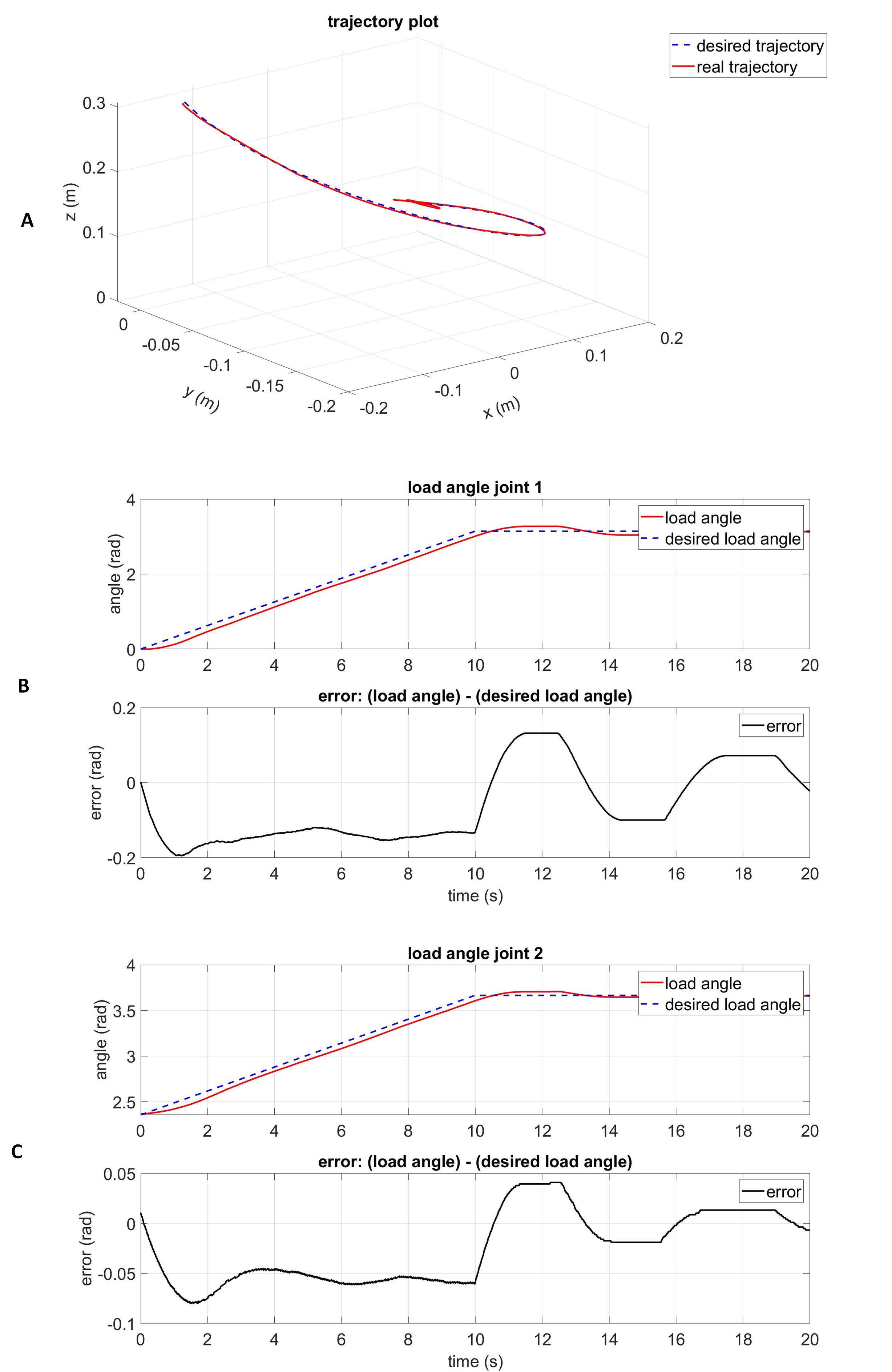

Supplement: Supplementary file 4 [file Image7.jpg]

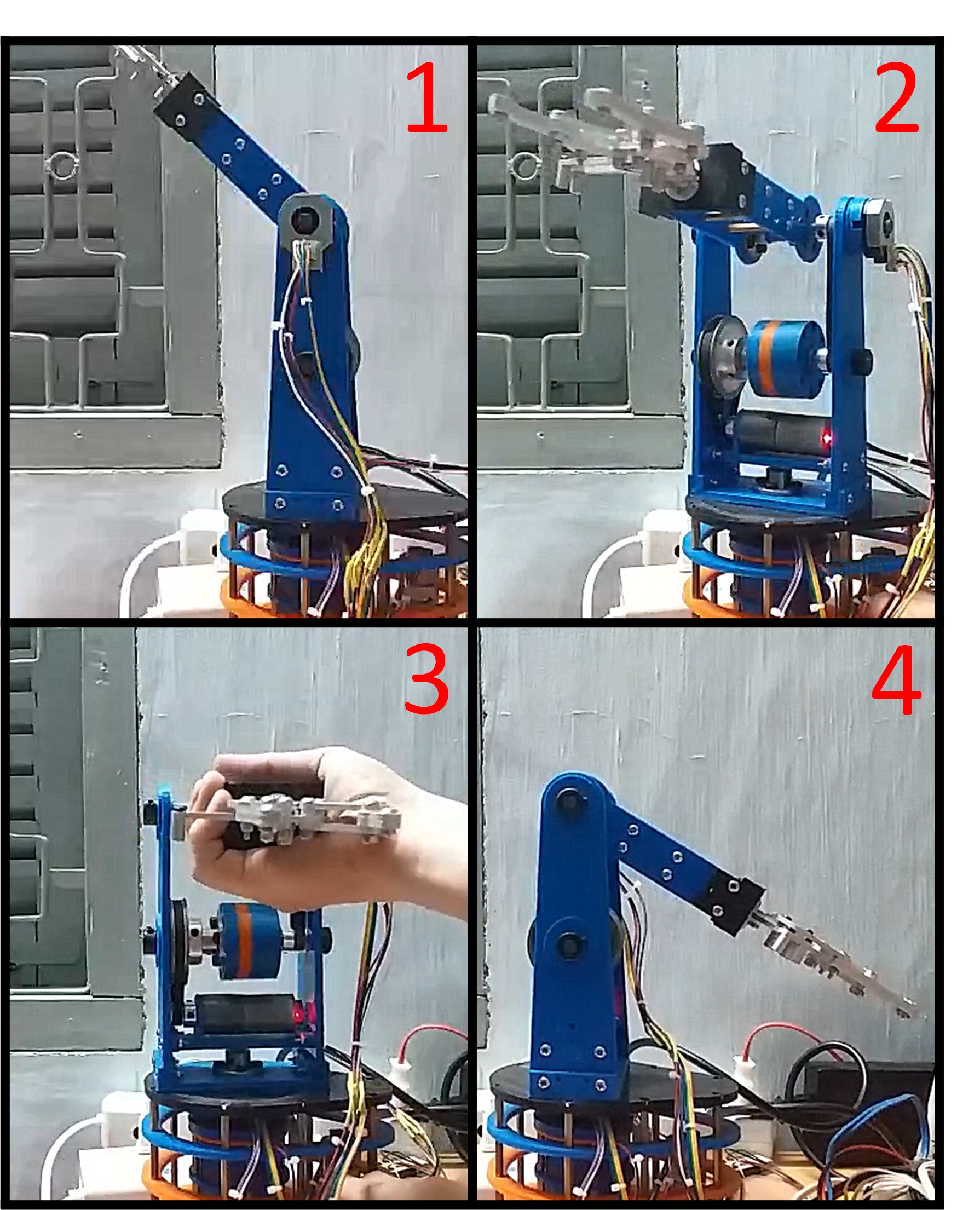

Supplement: Supplementary file 5 [file Image8.png]

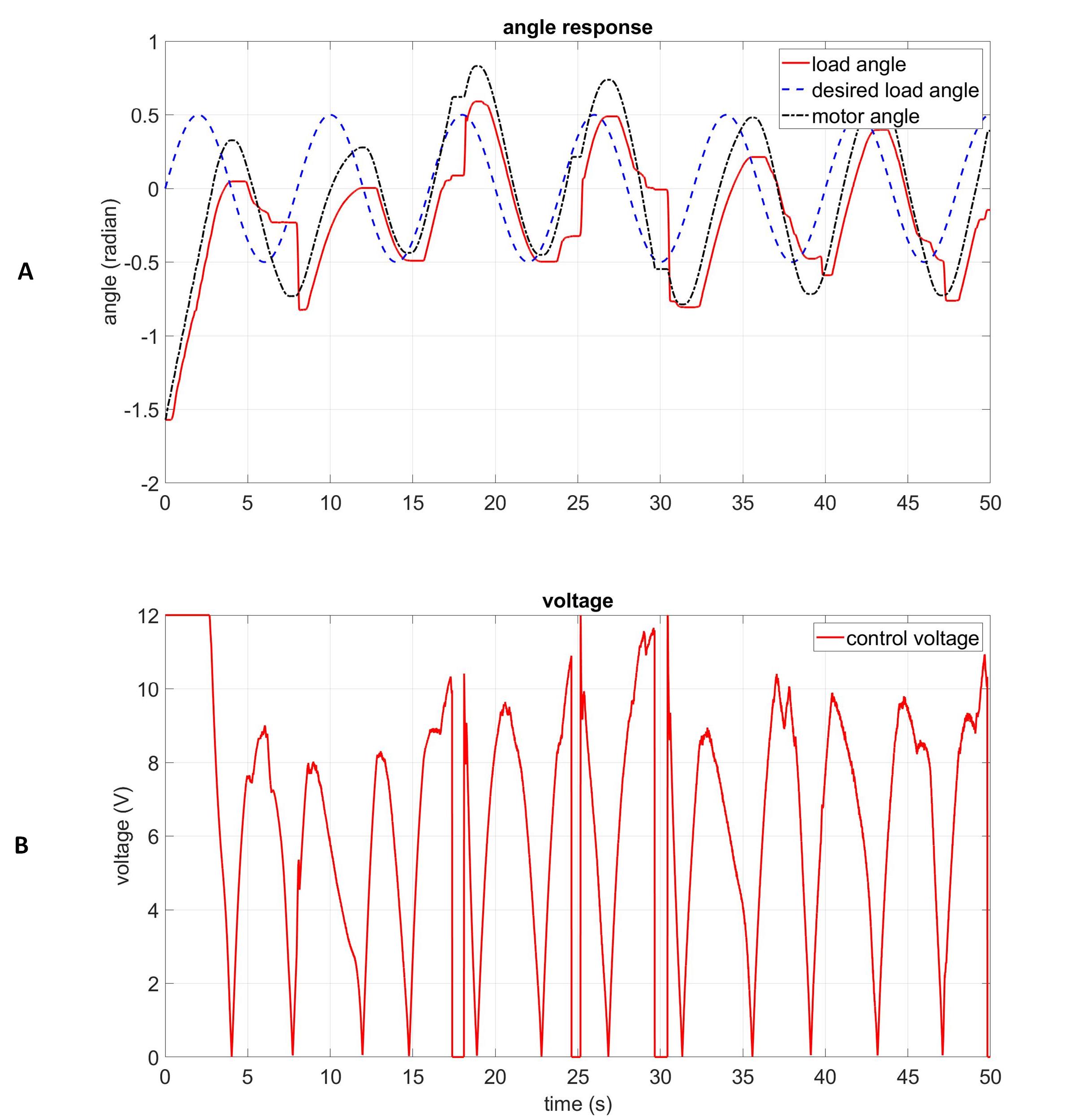

Supplement: Supplementary file 6 [file Image4.jpg]

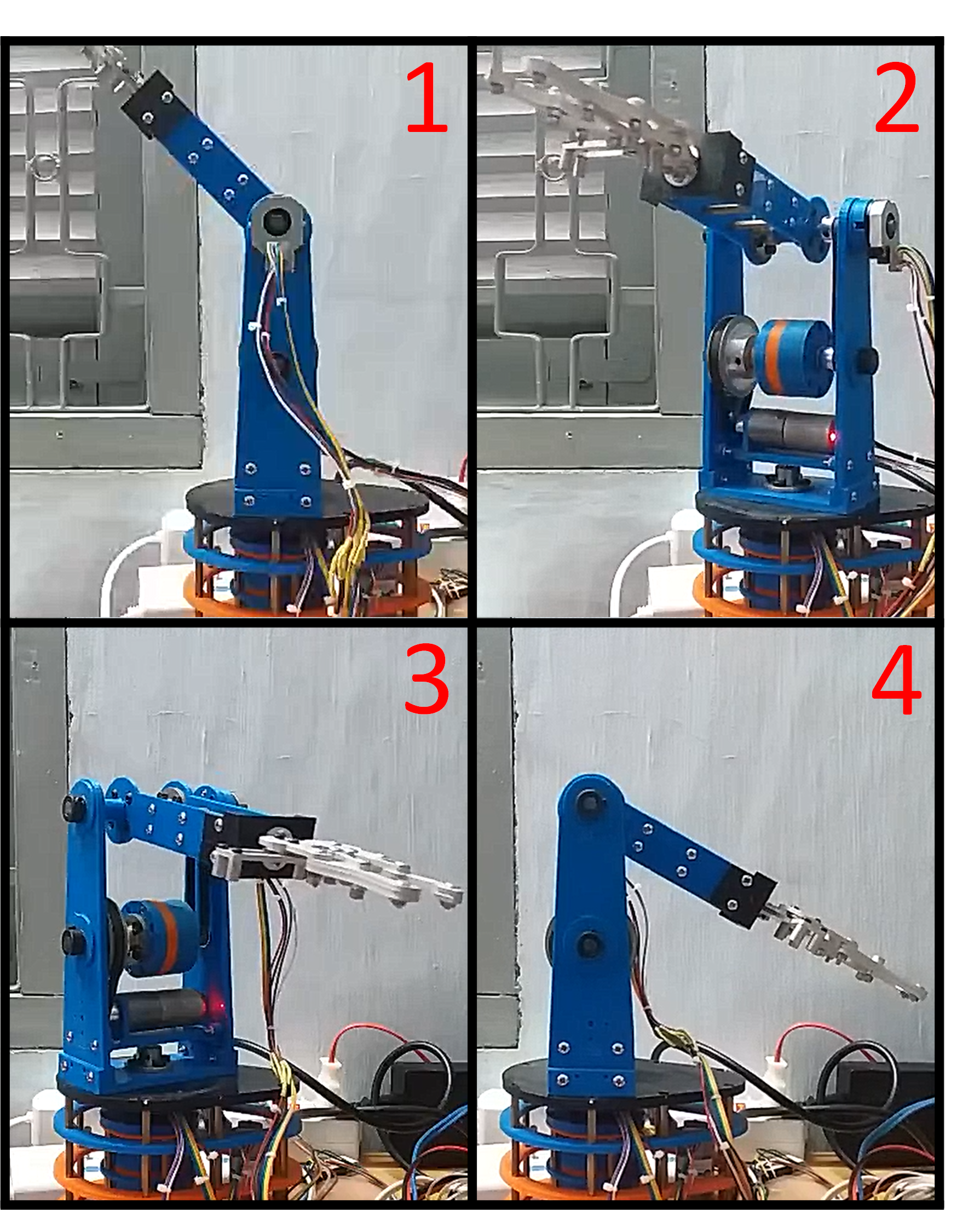

Supplement: Supplementary file 7 [file Image6.png]

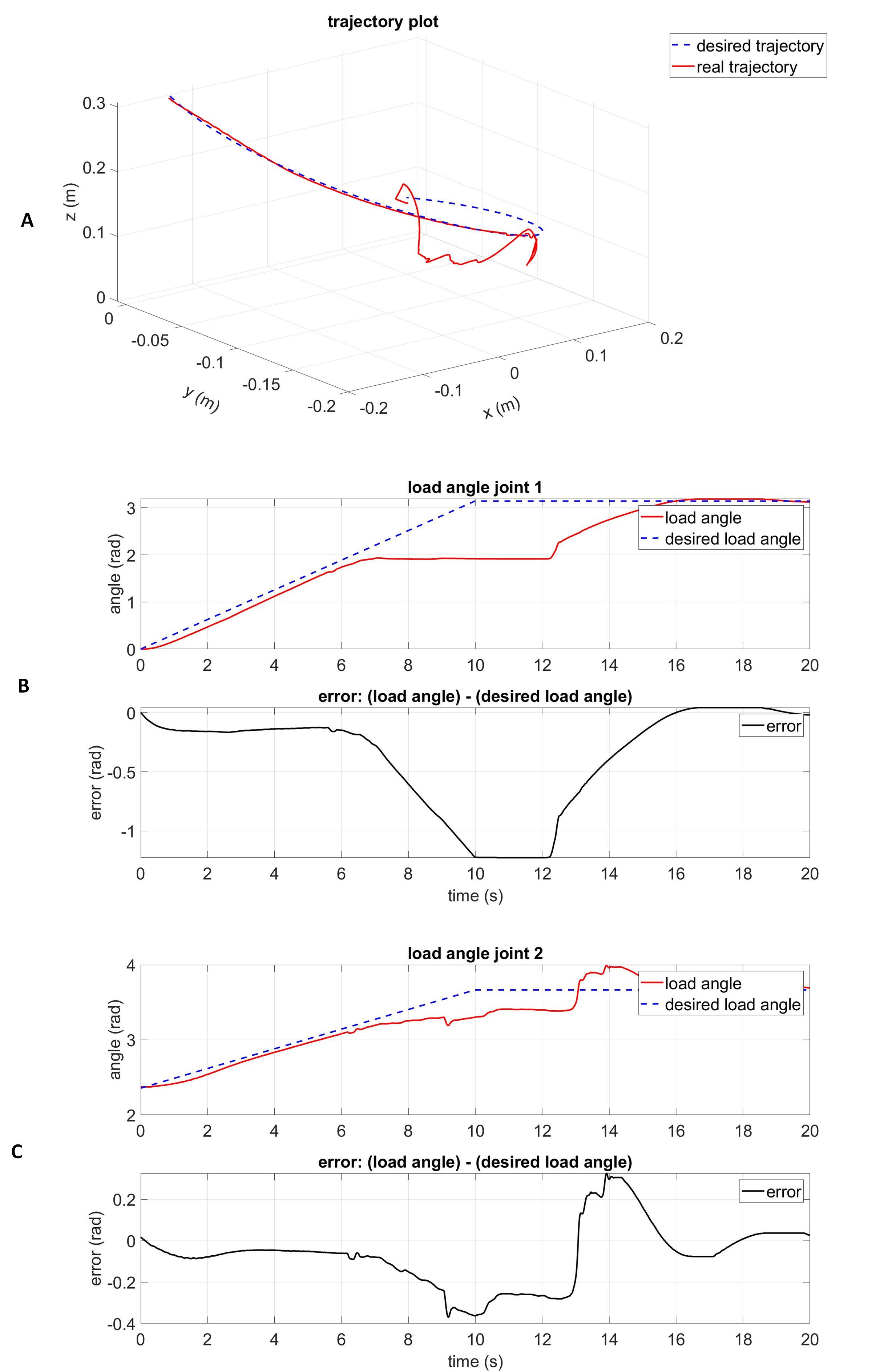

Supplement: Supplementary file 9 [file Image9.jpg]

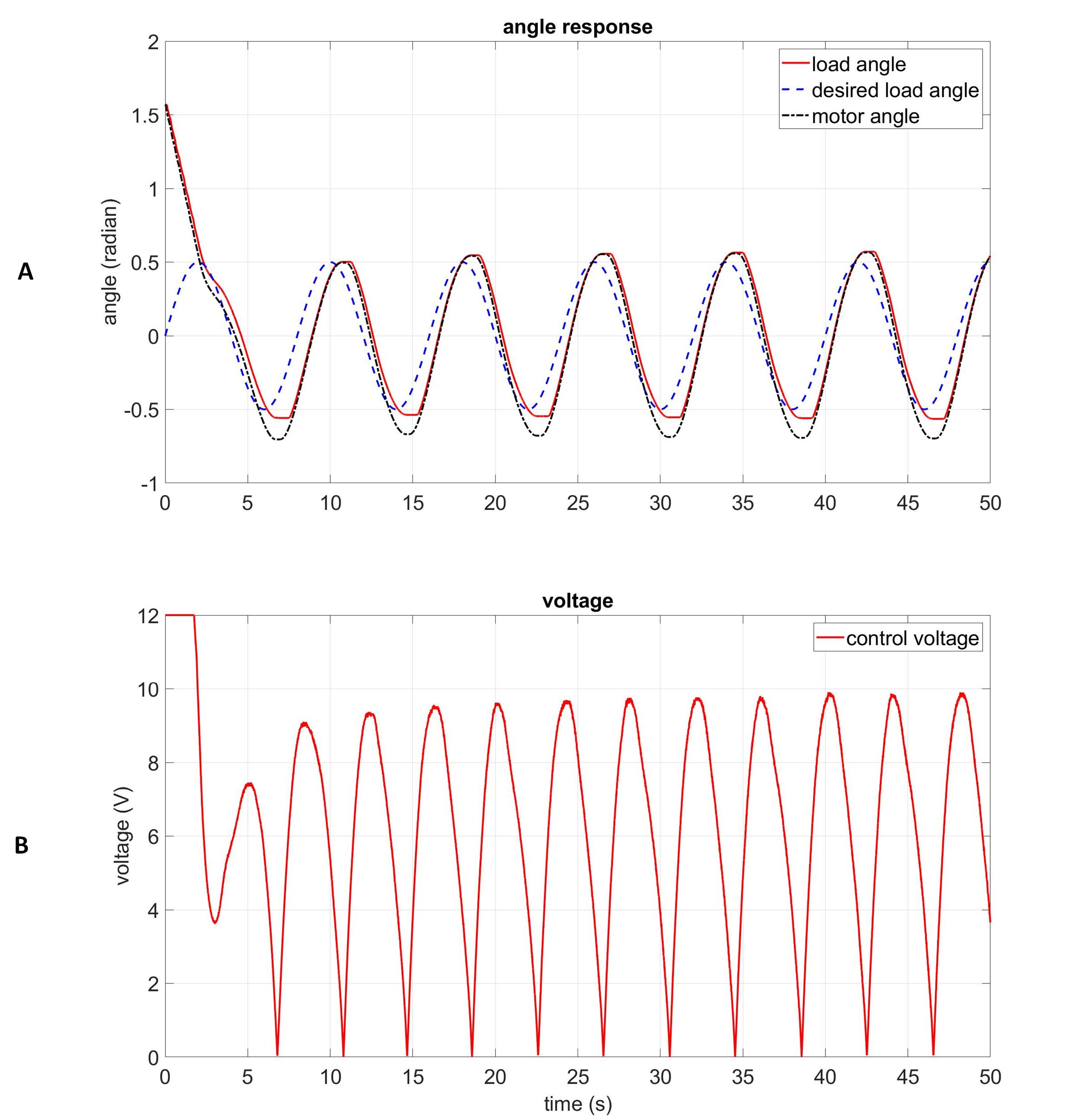

Supplement: Supplementary file 10 [file Image1.jpg]
